# Separable mechanisms drive local and global polarity establishment in the *Caenorhabditis elegans* intestinal epithelium

**DOI:** 10.1242/dev.200325

**Published:** 2022-11-16

**Authors:** Melissa A. Pickett, Maria D. Sallee, Lauren Cote, Victor F. Naturale, Deniz Akpinaroglu, Joo Lee, Kang Shen, Jessica L. Feldman

**Affiliations:** ^1^ Department of Biology, Stanford University, Stanford, CA 94305, USA; ^2^ Department of Biological Sciences, San Jose State University, San Jose, CA 95112, USA

**Keywords:** Apico-basolateral polarity, Par3/PAR-3, aPKC/PKC-3, Intestine, *Caenorhabditiselegans*

## Abstract

Apico-basolateral polarization is essential for epithelial cells to function as selective barriers and transporters, and to provide mechanical resilience to organs. Epithelial polarity is established locally, within individual cells to establish distinct apical, junctional and basolateral domains, and globally, within a tissue where cells coordinately orient their apico-basolateral axes. Using live imaging of endogenously tagged proteins and tissue-specific protein depletion in the *Caenorhabditiselegans* embryonic intestine, we found that local and global polarity establishment are temporally and genetically separable. Local polarity is initiated prior to global polarity and is robust to perturbation. PAR-3 is required for global polarization across the intestine but local polarity can arise in its absence, as small groups of cells eventually established polarized domains in PAR-3-depleted intestines in a HMR-1 (E-cadherin)-dependent manner. Despite the role of PAR-3 in localizing PKC-3 to the apical surface, we additionally found that PAR-3 and PKC-3/aPKC have distinct roles in the establishment and maintenance of local and global polarity. Taken together, our results indicate that different mechanisms are required for local and global polarity establishment *in vivo.*

## Teaser

**Highlighted Article:** Live-imaging and intestine-specific protein depletion in *Caenorhabditis elegans* reveal that apico-basolateral polarity establishment can be temporally and genetically separated locally, across individual cells, and globally, across a tissue.

## INTRODUCTION

Epithelial cells form adherent sheets that line organs, through which they separate internal and external compartments, act as barriers, selectively transport molecules and provide mechanical resilience. Underlying these functions is the characteristic polarization of epithelial cells along an apico-basolateral axis. The apical surface faces the external compartment or hollow lumen, whereas the basolateral domains provide contact between neighboring cells and attachment to underlying basement membranes. Adherens junctions (AJs) and occluding junctions [septate junctions (SJs) in invertebrates and tight junctions in vertebrates] are positioned between the apical and basolateral domains, where they provide cell-cell adhesion and barrier functions.

As epithelia are composed of individual polarized cells that collectively form a functional tissue, polarity establishment occurs both locally, with each individual cell establishing an apico-basolateral axis, and globally, such that neighboring cells coordinately orient their axes in the same direction. Polarity establishment at both levels is crucial for organ function. Loss of local cell polarity promotes cell growth in pre-invasive breast carcinomas (Halaoui et al., 2017), even though other cells in the tissue retain correct polarization. Partial or complete inversion of local polarity is associated with microvillus inclusion disease (Michaux et al., 2016), and pathogenic bacteria can appropriate local apico-basolateral polarity programs in the intestine, disrupting barrier function (Hua et al., 2018; Tapia et al., 2017a,b). Loss of global, tissue-level polarity results in non-adherent cells that disassociate or fail to undergo morphogenic movements, resulting in embryonic lethality (Achilleos et al., 2010; Bilder et al., 2000; Bossinger et al., 2001; Hutterer et al., 2004; Legouis et al., 2000; McMahon et al., 2001; Totong et al., 2007), or those that promote the progression and metastasis of cancers (Catterall et al., 2020; Ellenbroek et al., 2012; Halaoui et al., 2017; Zhou et al., 2017).

Despite the conservation of polarity proteins across species and epithelial tissues, the pathways by which these proteins are deployed and their requirement for polarity establishment are surprisingly divergent (Pickett et al., 2019). Canonically, in the best-understood polarity programs, the apical scaffolding protein PAR-3 (Par3 in mouse, Baz in *Drosophila*) is the most upstream known polarity player and is necessary to establish apical, junctional and basolateral domains (Achilleos et al., 2010; Harris and Peifer, 2004, 2005). Junctional proteins are required for refining polarity, and basolateral proteins play downstream roles in polarity maintenance (Bilder et al., 2000, 2003; Harris and Peifer, 2004; Legouis et al., 2000; McMahon et al., 2001). However, a holistic examination of polarity proteins at different stages in *Drosophila* blastoderm development has revealed that such polarity programs are more complex and less linear than they were originally thought to be. Baz acts both upstream and downstream of the AJ protein Canoe (AFD-1 in *C. elegans*, Afadin/Afdn in mouse) and the basolateral scaffolding proteins Dlg and Scribble were found to play essential roles in the initial positioning of AJs (Bonello et al., 2019, 2018). Other epithelia polarize independently of conserved polarity proteins or through parallel, redundant mechanisms, as in the *Drosophila* midgut and follicular epithelium, respectively (Chen et al., 2018; Fernandez-Minan et al., 2008; Pickett et al., 2019; Schneider et al., 2006; Tanentzapf et al., 2000). Thus, closer examination of how different epithelia polarize *in vivo* will provide a more complete understanding of polarity establishment pathways*.* As ubiquitous removal of polarity proteins from embryonic tissues often results in pleiotropic defects and embryonic lethality, tissue-specific protein depletion is crucial for understanding the range and requirement of different epithelial polarity programs.

We characterized the localization of polarity proteins relative to one another and degraded individual proteins specifically from the embryonic *Caenorhabditiselegans* intestine. The *C. elegans* intestine is a simple epithelium derived from a single blastomere (‘E’) that undergoes four rounds of division, giving rise to a 16-cell intestinal primordium (‘E16’) (Leung et al., 1999). At this E16 stage, ten dorsally positioned and six ventrally positioned cells surround a central midline, the future site of the apical surface of each cell and of the intestinal lumen (Leung et al., 1999). Proteins bound for the apical surface first concentrate locally as puncta on lateral membranes and then move to and spread along the central midline, beginning the specification of the global polarity axis in the intestine (Achilleos et al., 2010; Feldman and Priess, 2012; Totong et al., 2007). PAR-3 is the most upstream known polarity protein in the intestine, required for the apical localization of the PAR complex proteins PAR-6 and PKC-3 (Prkci in mouse, aPKC in *Drosophila*), and for establishing the junctions (Achilleos et al., 2010). Junctional and basolateral proteins are required for polarity maintenance, but when and how these proteins become organized into distinct domains is largely uncharacterized (Bossinger et al., 2004; Legouis et al., 2000; McMahon et al., 2001). Furthermore, whether local and global polarity depend on shared or separable mechanisms has not been investigated.

We found that local and global polarity establishment are separable processes in the *C. elegans* embryonic intestine. Local polarity establishment began prior to global establishment, with asymmetric localization of apical and AJ proteins into lateral puncta. In control embryos, global polarity appeared to arise in a stepwise manner with the apical surface established first, followed by the junctional structures and, lastly, the basolateral domain. However, intestine-specific depletion of polarity proteins revealed that polarity establishment is not a simple linear process. We found that PAR-3 and PKC-3 are both required for the formation of a functional intestine with a continuous, hollow lumen and for larval growth and viability, but that PAR-3 and PKC-3 play different roles in polarization. PKC-3 was not required to establish apico-basolateral polarity at the local or global levels but was required for SJ protein organization and for maintaining apical and junctional continuity. In contrast, PAR-3 was required for the initial establishment of polarity at both the local and global levels. Intriguingly, older PAR-3-depleted embryonic intestines contained small discontinuous regions with the correct relative organization of apical, junctional and basolateral proteins, indicating that local polarity was eventually established. These structures were lost when HMR-1 (E-cadherin/Cdh1 in mouse, Shg in *Drosophila*) was co-depleted with PAR-3, indicating that HMR-1 and PAR-3 redundantly drive local polarity establishment. Taken together, these results reveal that local polarity can eventually be established without PAR-3 but global polarity cannot, and thus local polarity establishment is robust and separable from global polarity establishment.

## RESULTS

### Apical and basolateral domains are established at different times and through different routes

**Fig. 1.**
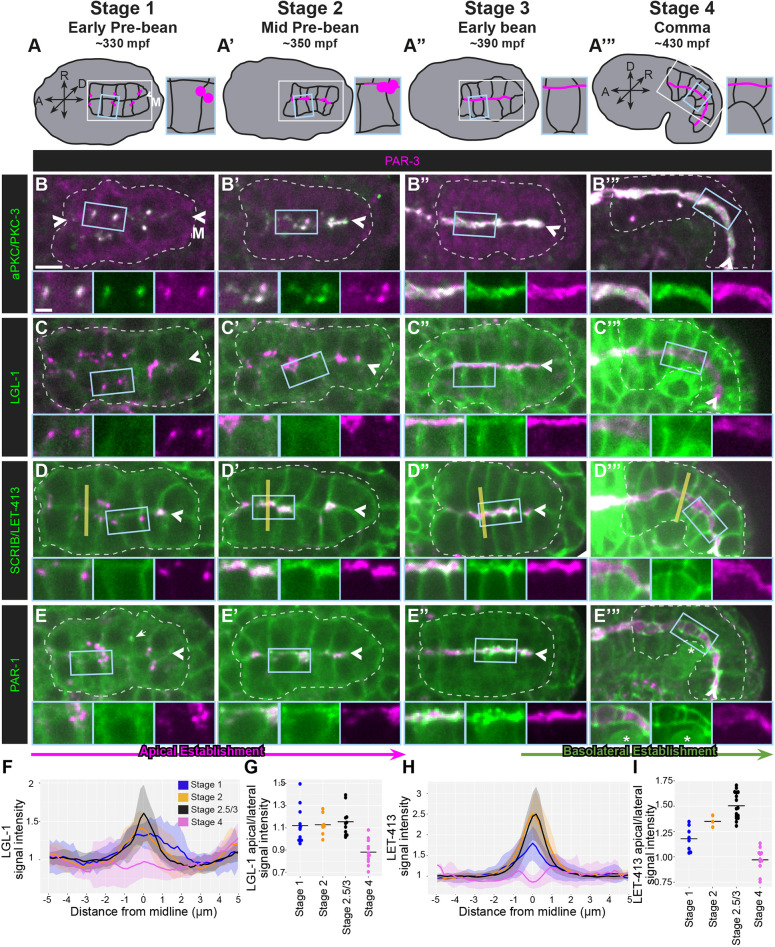
**Apical and basolateral proteins localize to the intestinal midline during global polarity establishment.** (A-A‴) Schematics of *C. elegans* embryonic morphogenesis from early pre-bean (stage 1) to comma (stage 4) stages, with minutes post-fertilization (mpf) indicated. Intestinal membranes are indicated in black lines, the intestines are marked with white boxes, and enlarged insets shown in the blue boxes. The body axis directions are indicated for embryos in A-A″. In A‴, the embryo has rotated onto its side, as depicted by the axes. ‘M’ indicates the midline. (B-E‴) Dorsal view of the colocalization of the indicated endogenously tagged proteins (green) and PAR-3 (magenta) in stage 1 (PKC-3, *n*=12; LGL-1, *n*=12 ; LET-413, *n*=11; PAR-1, *n*=10), stage 2 (PKC-3, *n*=11; LGL-1, *n*= 9; LET-413, *n*=8; PAR-1, *n*=12), stage 3 (PKC-3, *n*=5; LGL-1, *n*=9 ; LET-413, *n*=13; PAR-1, *n*=11) and stage 4 (PKC-3, *n*=16; LGL-1, *n*=12 ; LET-413, *n*=15; PAR-1, *n*=12) intestines. Note that PAR-1 occasionally localized to midbodies (E, arrow) in stage 1 and was highly expressed in germ cells (E‴, asterisk). All images are maximum-intensity projections from live imaging. Intestines are outlined by white dashed lines and the midlines indicated by arrowheads. Enlarged versions of the boxed regions are shown below each panel. Yellow lines in D-D‴ depict the approximate profiles drawn to quantify apical localization of LET-413 and LGL-1 in F,G. Scale bars: 5 µm (panels); 2 µm (magnified views). (F-I) Average line profiles for LGL-1 (F) or LET-413 (H) signal across the intestinal midline from stage 1 to stage 4, and quantification of the apical/lateral LGL-1 (G) or LET-413 (I) signal.

To systematically characterize the relative localization of polarity proteins throughout local and global polarity establishment, we performed live imaging for endogenously tagged apical, basolateral and junctional proteins in the *C. elegans* embryonic intestine. We restricted our analysis from the appearance of local polarity at the beginning of E16 through the comma stage, when the intestine is globally polarized (Achilleos et al., 2010; Beatty et al., 2010; Legouis et al., 2000). We found that four embryonic stages provided clear snapshots of local and global polarity establishment, which we defined based on embryo morphology and PAR-3 localization: (1) stage 1, early pre-bean, ∼330 min post fertilization (mpf); (2) stage 2, mid pre-bean, ∼350 mpf; (3) stage 3, early bean, ∼390 mpf; and (4) stage 4, comma, ∼430 mpf (Fig. 1A-A‴; Fig. S1).

Local polarity was initiated when the PAR complex proteins PAR-3 and PKC-3 colocalized in puncta on lateral cell membranes between adjacent non-sister cells (stage 1, Fig. 1B), consistent with previous observations (Achilleos et al., 2010; Feldman and Priess, 2012). These ‘lateral puncta’ moved toward the intestinal midline, where they appeared to spread within each cell (stage 2, Fig. 1B′; Movies 1, 2 and 4), creating a continuous apical surface along the intestinal midline and establishing global apical polarity (stage 3, Fig. 1B″) that persisted throughout intestinal morphogenesis (stage 4, Fig. 1B‴).

In contrast, basolateral proteins first localized to all plasma membranes, including the apical membrane, before becoming restricted in their localization. LGL-1 [Lgl in mouse, L(2)gl in *Drosophila*] and LET-413 (Scribble/Scrib in mouse and *Drosophila*) initially localized to all cell membranes and were neither localized to nor excluded from the lateral puncta (stage 1, Fig. 1C,D,F-I; Movie 1), becoming more strongly associated with all plasma membranes as polarization progressed (stage 2, Fig. 1C′,D′,F-I). Surprisingly, LGL-1 and LET-413 appeared to colocalize with PAR-3 at the nascent apical surface (stage 3, Fig. 1C″,D″,F-I), although there may have been some separation of proteins beyond the resolution of our microscope. Both LGL-1 and LET-413 were excluded by stage 4, as previously described (Fig. 1C‴,D‴,F-I) (Beatty et al., 2010; Bossinger et al., 2004; Legouis et al., 2000).

We also examined the localization of the conserved kinase PAR-1 (MARK2 in mouse, Par1 in *Drosophila*), which localizes to basolateral membranes in many epithelia. We tagged endogenous PAR-1 with GFP and found that it localized to the centrosomes during mitosis prior to stage 1 and occasionally at the midbodies in stage 1 embryos (Fig. 1E), consistent with the ability of PAR-1 to bind to microtubules and associate with microtubule-binding proteins (Cohen et al., 2004; Doerflinger et al., 2003; Sanchez et al., 2021). At stage 1, PAR-1 localized weakly to plasma membranes (Fig. 1E), similar to LGL-1 and LET-413. Unlike LGL-1 or LET-413, PAR-1 separated from PAR-3 in stage 2 (Fig. 1E′; Movie 2). PAR-1 then localized into subapical bands, losing its lateral localization (stage 3, Fig. 1E″), and organized into ladder-like junctions by stage 4 (Fig. 1E‴). Thus, PAR-1 shows junctional rather than basolateral localization in the *C. elegans* intestine.

### Adherens and septate junction proteins localize separately during polarity establishment

**Fig. 2.**
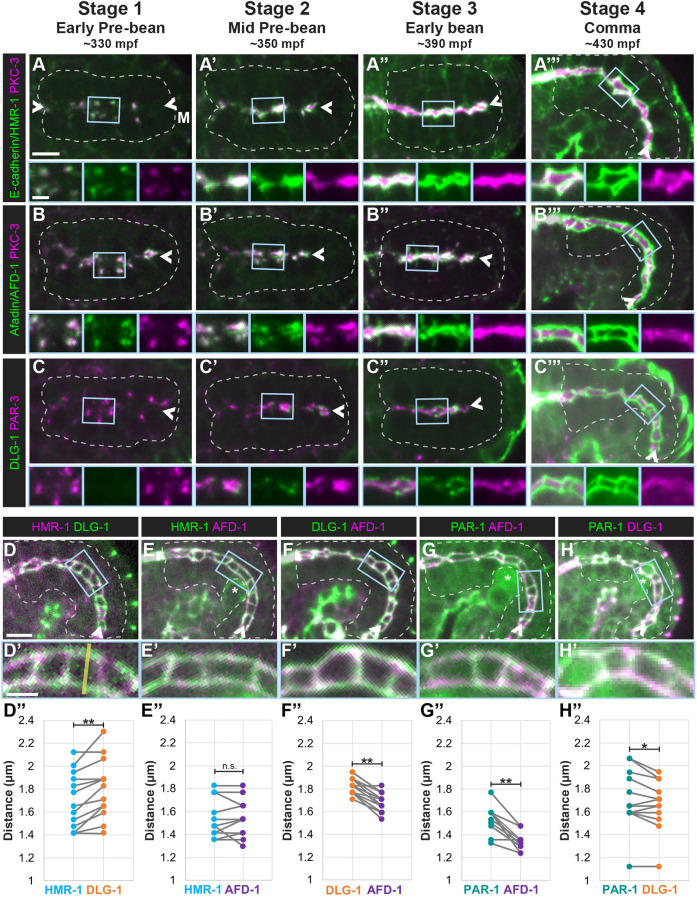
**Adherens and septate-like junctional proteins localize at different times and places during local and global polarity establishment.** (A-C) Dorsal view of colocalization of the indicated endogenously tagged proteins (green) and either PKC-3 (A,B, magenta) or PAR-3 (C, magenta) in live stage 1 (HMR-1, *n*=11; AFD-1, *n*=8; DLG-1, *n*=14), stage 2 (HMR-1, *n*=13; AFD-1, *n*=26; DLG-1, *n*=13), stage 3 (HMR-1, *n*=11; AFD-1, *n*=8; DLG-1, *n*=17) and stage 4 (HMR-1, *n*=5; AFD-1, *n*=9; DLG-1, *n*=12) intestines. (D-H) Dorsolateral views of the indicated endogenously tagged proteins in live stage 5 (1.5-fold) embryos (D, *n*=15; E, *n*=12; F, *n*=15; G, *n*=12; H, *n*=14). Asterisks mark germ cells. Magnified views of the blue boxed regions are shown below. All images are maximum-intensity projections from live imaging. Intestines are outlined by white dashed lines and the midlines are indicated by arrowheads. Scale bars: 5 µm (panels); 2 µm (magnified views). (D″-H″) Quantification of the distance between the left and right sides of the HMR-1 and DLG-1 junctional structures, measured with a line profile for signal intensity as shown by the yellow line in D′ (two-tailed paired *t*-test; n.s., not significant; *P=0.0.0216; **P<0.001).

We next examined the localization of junctional proteins. In the mature *C. elegans* digestive tract, AJ and SJ proteins occupy separate domains of a single ‘apical junction’ (Köppen et al., 2001; Segbert et al., 2004). We therefore tested whether AJ and SJ proteins showed similar or distinct localization patterns during polarity establishment. Consistent with previous studies (Achilleos et al., 2010; Totong et al., 2007), we found that the AJ protein HMR-1 colocalized with PKC-3 in lateral puncta in stage 1 intestines, and both moved together toward (Fig. 2A) and subsequently spread along the midline (stage 2, Fig. 2A′). Shortly after spreading across the midline, HMR-1 shifted into bands parallel to the apical surface (stage 3, Fig. 2A″) forming the characteristic ladder-like junctions in older embryos (stage 4, Fig. 2A‴). We next explored the localization of AFD-1, a conserved AJ protein that is required for Baz localization in the *Drosophila* blastoderm (Bonello et al., 2018; Choi et al., 2013), which was uncharacterized in the *C. elegans* intestine. We tagged endogenously expressed AFD-1 with GFP and found that similar to HMR-1, AFD-1 localized to lateral puncta that moved toward the midline (stages 1 and 2, Fig. 2B,B′; Movie 3), before separating into subapical bands (stage 3, Fig. 2B″) and organizing into junctions (stage 4, Fig. 2B‴).

Differences in AJ and SJ protein localization were apparent at the onset of polarity establishment in stage 1 as the SJ protein DLG-1 was not observed (Fig. 2C; Movie 4), consistent with prior observations (Achilleos et al., 2010; Totong et al., 2007). In stage 2 intestines, DLG-1 localized in subapical puncta that were adjacent to but not colocalized with PAR-3 (Fig. 2C′; Movie 4). DLG-1 then formed fragmented subapical bands adjacent to the apical surface (stage 3, Fig. 2C″), which appeared less continuous and more basal than the localization of HMR-1 and AFD-1 at the same stage. These bands eventually formed junctions (stage 4, Fig. 2C‴).

To elaborate how AFD-1 and PAR-1 proteins are organized within the mature junctions, we compared the localization of junctional proteins in 1.5-fold- to 1.7-fold-stage embryos. We measured the distance across the midline between intensity peaks, corresponding to the left and right sides of the junction, for two junctional markers, pairing the measurements for each embryo to account for inter-embryo variation in junctional width (see Materials and Methods; Fig. S2). Confirming the sensitivity of our method, we measured a significantly smaller distance between intensity peaks of the AJ protein HMR-1 than that of the SJ protein DLG-1 (Fig. 2D-D″), consistent with HMR-1 localizing apical to DLG-1 as previously shown (Segbert et al., 2004). The distance between AFD-1 peaks did not differ from that of HMR-1 (Fig. 2E-E″), but was significantly smaller than that of DLG-1 (Fig. 2F-F″), consistent with AFD-1 localizing to the AJ-like region of the apical junction. Surprisingly, the PAR-1 distance was significantly greater than both AFD-1 (Fig. 2G-G″) and DLG-1 (Fig. 2H-H″) distances. The junctional component of LET-413 similarly had a significantly greater distance than that of DLG-1 (Fig. 5E), suggesting that PAR-1 and LET-413 may localize to a more basal region of the apical junction. These analyses extend previous findings (Segbert et al., 2004), with proteins falling into distinct regions with HMR-1 and AFD-1 showing the most apical localization, followed by DLG-1, and PAR-1 and LET-413 being the most basal.

The differences in the localization and movement of polarity proteins into distinct domains indicate that multiple mechanisms likely contribute to local and global polarity establishment. Additionally, the separation of junctional structures from the apical surface when basolateral proteins are still colocalized with PAR-3 suggests that global apico-basolateral polarity proceeds temporally in a stepwise manner with the apical surface established first, followed by establishment of the junctions, and lastly of the basolateral domain.

### Different apical and junctional proteins play distinct roles in intestine formation and organismal health

**Fig. 3.**
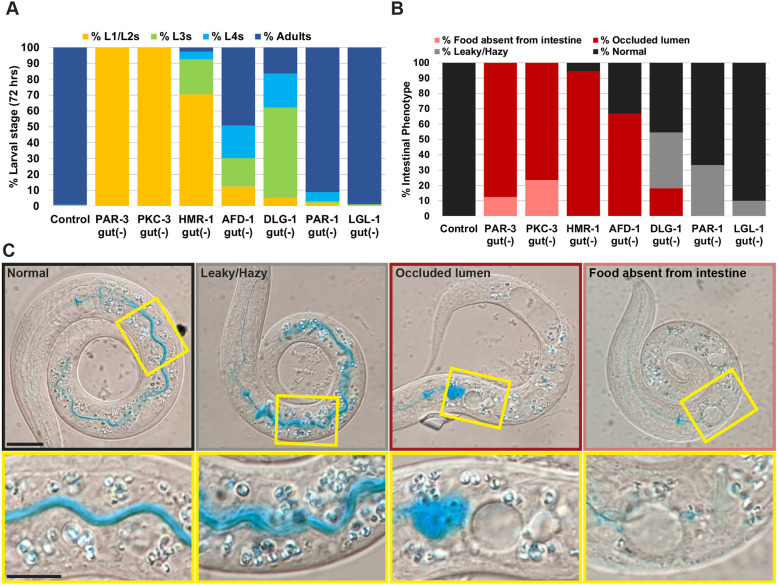
**Polarity proteins are differentially required for intestinal structure and function and for larval growth.** (A) Percentage of worms at the L1/L2, L3 or L4 larval or adult stages 72 h after egg lay for control [*zif-1(gk117);intDeg*] or intestine-specific depletion of the indicated proteins (control, *n*=696; PAR-3^gut(−)^, *n*=198; PKC-3^gut(−)^, *n*=132; HMR-1^gut(−)^, *n*=186; AFD-1^gut(−)^, *n*=146; DLG-1^gut(−)^, *n*=116; PAR-1^gut(−)^, *n*=171; LGL-1^gut(−)^, *n*=269). 100% of PAR-3^gut(−)^ and PKC-3^gut(−)^ worms arrested as L1 larvae. (B) Percentage of L1 larvae with normal, leaky/hazy or occluded intestinal lumens, or entirely lacking food in the intestine for control or following intestine-specific depletion of the indicated proteins (control, *n*=13; PAR-3^gut(−)^, *n*=16; PKC-3^gut(−)^, *n*=17; HMR-1^gut(−)^, *n*=18; AFD-1^gut(−)^, *n*=12; DLG-1^gut(−)^, *n*=11; PAR-1^gut(−)^, *n*=15; LGL-1^gut(−)^, *n*=10). (C) Representative DIC images of live worms fed blue food coloring showing the indicated phenotypic categories, with higher-magnification views of the boxed regions shown below. Scale bars: 20 µm (panels); 10 µm (magnified views).

Strategies for the ubiquitous depletion of proteins have identified proteins required for polarity establishment or maintenance in the *C. elegans* intestine (Achilleos et al., 2010; Bossinger et al., 2004, 2001; Firestein and Rongo, 2001; Köppen et al., 2001; Legouis et al., 2000; McMahon et al., 2001; Segbert et al., 2004; Totong et al., 2007; Von Stetina and Mango, 2015). However, the essential roles of these proteins in many tissues often resulted in embryonic lethality when depleted, which precluded further functional analysis. Therefore, we depleted polarity proteins specifically from the intestine using the ZF/ZIF-1 system (Armenti et al., 2014; Sallee et al., 2018). Using CRISPR/Cas9, we endogenously tagged polarity proteins with ZF:GFP, allowing ZIF-1-mediated degradation via the ZF1 degron tag (Nance et al., 2003) (Figs S3 and S4). We expressed ZIF-1 under the *elt-2* promoter for efficient intestine-specific protein depletion [‘gut(−)’] beginning in embryos at the four-cell intestinal stage (‘E4’), well before polarization, and continuing through adulthood. Intestine-specific depletion of either PAR-3 or PKC-3 [PAR-3^gut(−)^ or PKC-3^gut(−)^] resulted in 100% developmental arrest at the first larval stage (‘L1’) (Fig. 3A). Similarly, intestine-specific depletion of HMR-1 caused L1 larval arrest, although ∼30% of HMR-1^gut(−)^ larvae grew more slowly and developed beyond the L1 stage (Fig. 3A). Larvae depleted of other junctional proteins, e.g. AFD-1^gut(−)^ or DLG-1^gut(−)^, also grew slower than control larvae; although, unlike HMR-1^gut(−)^, most developed beyond the L1 stage.

To determine whether intestinal structure and function were compromised following the depletion of apical or junctional proteins, we fed newly hatched L1 larvae bacteria dyed with blue food coloring (‘Smurf assay’), the localization of which is normally limited to the continuous, hollow intestinal lumen (Fig. 3B,C) (Gelino et al., 2016; Sallee et al., 2021). The majority of PAR-3^gut(−)^ or PKC-3^gut(−)^ larvae showed occluded intestinal lumens, with food pooled posterior to the pharynx and large edemas throughout the intestine (Fig. 3B,C), as we observed previously in PAR-6^gut(−)^ and PKC-3^gut(−)^ larvae (Sallee et al., 2021). Most HMR-1^gut(−)^ L1 larvae also had anterior intestinal occlusions (88%) and discontinuous edematous lumens (72%, Fig. 3B,C; Fig. S5). However, many HMR-1 L1 larvae had regions of continuous lumens after the anterior occlusion (66%). The majority of AFD-1^gut(−)^ worms exhibited anterior occlusions (58%, Fig. 3B,C; Fig. S5); however, food filled more of the anterior intestine than in PAR-3^gut(−)^, PKC-3^gut(−)^ or HMR-1^gut(−)^ larvae and the lumens appeared to be continuous posterior to the occlusion (Fig. S5). Most DLG-1^gut(−)^ larvae had a continuous lumen by differential interference contrast (DIC) microscopy and the Smurf assay (75%, Fig. 3B,C), although we often observed the blue dye outside the intestinal lumen, perhaps indicating barrier dysfunction (36% leaky/hazy, Fig. 3B,C; Fig. S5). PAR-1^gut(−)^ larvae had still milder defects, with only ∼9% showing a developmental delay (Fig. 3A) and only 33% of PAR-1^gut(−)^ L1 larval intestines showing possible barrier dysfunction (Fig. 3B; Fig. S5). Intestine-specific depletion of the basolateral protein LGL-1 did not slow worm growth or perturb intestinal function (Fig. 3A-C), consistent with earlier studies showing that LGL-1 is dispensable for worm growth and survival (Beatty et al., 2010). Taken together, these results reveal differences in protein requirement to build a functional intestine, with the apical PAR and AJ proteins crucial for intestinal function and organismal viability, and SJ and basolateral proteins relatively dispensable.

### Apical and junctional proteins play different roles in global apical establishment

We next asked how depletion of different apical and junctional proteins affected polarity establishment. We found that PAR-3 was required to localize PKC-3 at all stages of polarity establishment (Fig. S6), consistent with prior studies and indicative of effective PAR-3 degradation (Achilleos et al., 2010). PKC-3 was not required for initial PAR-3 localization (Fig. S6), but was required to maintain continuous PAR-3 localization along the midline, as gaps appeared along this surface in elongating embryos, similar to PAR-6^gut(−)^ embryos (Fig. S6) (Sallee et al., 2021; Totong et al., 2007). Consistent with the mild defects observed in AFD-1^gut(−)^ L1 larvae, AFD-1^gut(−)^ embryos had relatively normal apical polarity compared to PAR-3 depleted intestines. Stage 1 AFD-1^gut(−)^ embryos had fewer PAR-3 and PKC-3 lateral puncta, particularly in the anterior part of the intestine, and generally reduced PKC-3 fluorescence intensity (Fig. S6). The defect in the anterior intestinal cells persisted through stage 2 (Fig. S6), although PKC-3 became correctly localized to the midline beginning in stage 3. Minor extensions of PKC-3 away from the apical surface were occasionally observed in stage 4 and older AFD-1^gut(−)^ embryos (Fig. S6), similar to observations in embryos with actin defects (Gobel et al., 2004; Ramalho et al., 2020; Sanchez et al., 2021; Sasidharan et al., 2018; Van Furden et al., 2004). Importantly, this result suggests that although AFD-1 may contribute to the assembly and movement of lateral puncta to the intestinal midline, other mechanisms are sufficient for local and global apical establishment. By contrast, PAR-3 and PKC-3 lateral puncta formed and moved to the intestinal midline normally in PAR-1^gut(−)^ embryos (Fig. S6). In stage 4 and older PAR-1^gut(−)^ embryos, we occasionally observed a broader distribution of apical proteins across the midline of the intestine (Fig. S6).

### PAR-3 has PKC-3-independent roles in the positioning and maturation of junctions

**Fig. 4.**
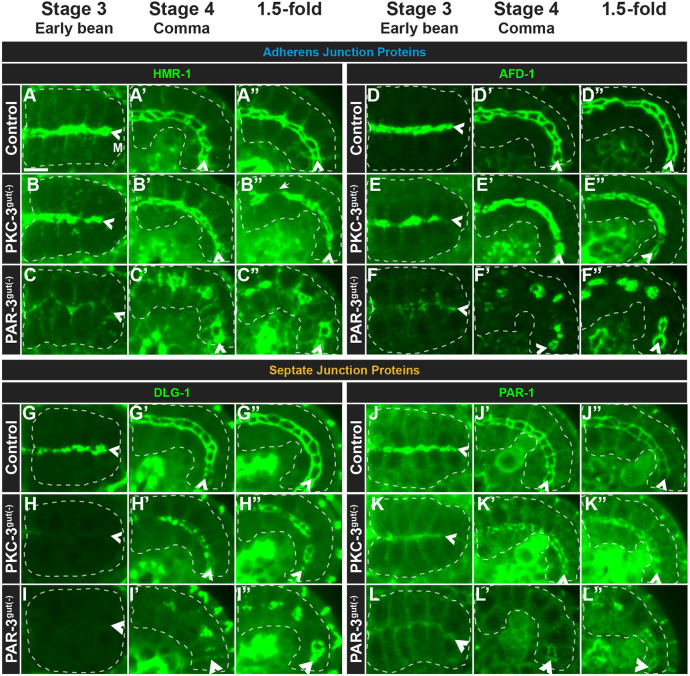
**PAR-3 and PKC-3 play different roles in AJ and SJ protein localization.** (A-L″) Dorsal (stage 3) or dorsolateral (stage 4 and 1.5-fold) live images of the indicated endogenously tagged AJ or SJ proteins in control (A,D,G,J), PKC-3^gut(−)^ (B,E,H,K) or PAR-3^gut(−)^ (C,F,I,L) embryos. (B″) Gaps in junctional proteins frequently appeared in 1.5-fold PKC-3^gut(−)^ intestines (B″, arrow). All images are maximum-intensity projections from live imaging. Intestines are outlined by white dashed lines and the midlines are indicated by arrowheads. Scale bar: 5 µm. Stage 3 HMR-1 (control, *n*=14; PAR-3^gut(−)^, *n*=10; PKC-3^gut(−)^, *n*=13); AFD-1 (control, *n*=4; PAR-3^gut(−)^, *n*=3; PKC-3^gut(−)^, *n*=6); DLG-1 (control, *n*=12; PAR-3^gut(−)^, *n*=1; PKC-3^gut(−)^, *n*=7); PAR-1 (control, *n*=4; PAR-3^gut(−)^, *n*=2; PKC-3^gut(−)^, *n*=9). Stage 4 HMR-1 (control, *n*=8; PAR-3^gut(−)^, *n*=18; PKC-3^gut(−)^, *n*=12); AFD-1 (control, *n*=10; PAR-3^gut(−)^, *n*=7; PKC-3^gut(−)^, *n*=12); DLG-1 (control, *n*=24; PAR-3^gut(−)^, *n*=12; PKC-3^gut(−)^, *n*=26); PAR-1 (control, *n*=13; PAR-3^gut(−)^, *n*=16; PKC-3^gut(−)^, *n*=16). 1.5-fold HMR-1 (control, *n*=5; PAR-3^gut(−)^, *n*=12; PKC-3^gut(−)^, *n*=13); AFD-1 (control, *n*=6; PAR-3^gut(−)^, *n*=8; PKC-3^gut(−)^, *n*=10); DLG-1 (control, *n*=23; PAR-3^gut(−)^, *n*=5; PKC-3^gut(−)^, *n*=19); PAR-1 (control, *n*=8; PAR-3^gut(−)^, *n*=10; PKC-3^gut(−)^, *n*=8).

Given the similarity of the terminal phenotype of PAR-3^gut(−)^ and PKC-3^gut(−)^ larvae, we asked whether PAR-3 and PKC-3 play similar roles in embryonic intestinal development. PAR-6, PAR-3 and PKC-3 homologs play roles in junctional establishment and organization in many systems; thus, we determined whether PAR-3 regulates junctions through PKC-3 activity or through a separate mechanism by examining the localization of junctional proteins in PKC-3^gut(−)^ or PAR-3^gut(−)^ embryos (Achilleos et al., 2010; Elbediwy et al., 2019; Iden et al., 2012; Sallee et al., 2021; Totong et al., 2007). HMR-1 and AFD-1 localized near the newly established apical surface along the intestinal midline in both stage 3 control (Fig. 4A,D) and PKC-3^gut(−)^ embryos (Fig. 4B,E). In stage 4 and 1.5-fold control embryos, HMR-1 and AFD-1 localized into subapical junctions (Fig. 4A′,A″,D′,D″), but these junctions were closer together in 1.5-fold PKC-3^gut(−)^ embryos, possibly reflecting a narrower apical surface or inefficient junctional separation (Fig. 4B′,E′; Fig. S7). In 1.5-fold PKC-3^gut(−)^ embryos, we observed gaps in HMR-1 and AFD-1 localization (Fig. 4B″,E″), indicating that PKC-3 is not initially required to organize AJ proteins but is required for the growth and continuity of the AJs. By contrast, in stage 3 PAR-3^gut(−)^ embryos, HMR-1 and AFD-1 localized weakly in patches and along membranes (Fig. 4C,F) instead of as parallel subapical bands. In stage 4 PAR-3^gut(−)^ embryos, HMR-1 and AFD-1 formed separated rings (Fig. 4C′,F′), which became more continuous and prominent in the intestines of 1.5-fold embryos (Fig. 4C″,F″). Surprisingly, HMR-1 and AFD-1 were excluded from the centers of the rings (Fig. 4C″,F″), suggesting that local junction organization may be intact within small cell clusters. The differences in AJ localization between PAR-3^gut(−)^ and PKC-3^gut(−)^ embryos indicate that PAR-3 and PKC-3 have separable roles in AJ protein localization.

**Fig. 5.**
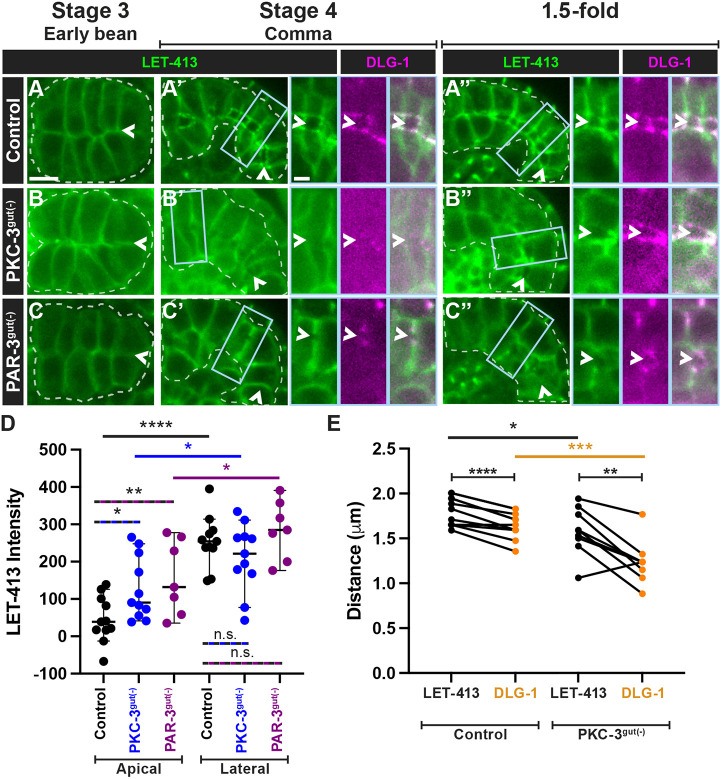
**PAR-3 and PKC-3 are not required for apical exclusion of LET-413.** (A-C) Dorsal (stage 3) and dorsolateral (stage 4 and 1.5-fold) live images of endogenously tagged LET-413 (green) in control (A-A″), PAR-3^gut(−)^ (B-B″) or PKC-3^gut(−)^ (C-C″) embryos. Embryos in A′-C″ co-express endogenously tagged DLG-1 (magenta), with magnified views of the boxed regions shown on the right. All images are maximum-intensity projections from live imaging. Intestines are outlined by white dashed lines and the midlines are indicated by arrowheads. Scale bar: 5 µm (panels); 2 µm (magnified views). Stage 3 (control, *n*=5; PAR-3^gut(−)^, *n*=7; PKC-3^gut(−)^, *n*=8); stage 4 (control, *n*=11; PAR-3^gut(−)^, *n*=5; PKC-3^gut(−)^, *n*=11); 1.5-fold (control, *n*=11; PAR-3^gut(−)^, *n*=8; PKC-3^gut(−)^, *n*=15). (D) Scatter dot plots of the apical and lateral LET-413 signal intensity (signal−cytoplasmic signal) for control (n=11), PKC-3^gut(−)^ (n=11) and PAR-3^gut(−)^ (n=7) 1.5-fold embryos. Data show the median with 95% c.i. Two-tailed unpaired Student's *t*-test was used to determine significance. (E) Quantification of the distance between the LET-413 and DLG-1 junctional structures in control (n=11) and PKC-3^gut(−)^ (n=9) 1.5-fold embryos. Two-tailed paired Student's *t*-test was used for LET-413 versus DLG-1 distance in control or PKC-3^gut(−)^, and two-tailed unpaired Student's *t*-test was used for control versus PKC-3^gut(−)^. n.s., not significant; *P<0.05; **P<0.01; ***P<0.001; ****P<0.0001.

We next explored whether SJ proteins were disrupted upon PAR-3 or PKC-3 depletion. In control embryos, PAR-1 and DLG-1 localized in subapical bands in the intestines of stage 3 embryos and localized to the junctions in stage 4 and 1.5-fold embryos (Fig. 4G-G″,J-J″). In the intestines of stage 3 PKC-3^gut(−)^ embryos, DLG-1 localization was absent and PAR-1 appeared to be weakly associated with the apical and lateral cell membranes, but was absent from subapical bands (Fig. 4H,K). In stage 4 PKC-3^gut(−)^ embryos, both DLG-1 and PAR-1 localized weakly and discontinuously into subapical junctions (Fig. 4H′,K′) and remained discontinuous in subapical bands in the intestines of 1.5-fold embryos (Fig. 4H″,K″), suggesting that PKC-3 is required for the correct timing and organization of SJ proteins. As with the AJ proteins, the distance across the midline between SJ proteins on apposing cells was significantly smaller in PKC-3^gut(−)^ 1.5-fold embryos (Fig. 5E; Fig. S7).

In the intestines of stage 3 PAR-3^gut(−)^ embryos, DLG-1 failed to localize (Fig. 4I) and PAR-1 localized weakly to the membrane (Fig. 4L). DLG-1 first localized in discontinuous patches throughout the intestine of stage 4 PAR-3^gut(−)^ embryos (Fig. 4I′) and later organized into rings in 1.5-fold embryos (Fig. 4I″). PAR-1 also localized in rings, first primarily in the posterior regions of the intestine in stage 4 PAR-3^gut(−)^ embryos (Fig. 4L′) and then throughout the intestine in 1.5-fold embryos (Fig. 4L″). The PAR-1 and DLG-1 structures resembled the HMR-1 and AFD-1 rings described above but assembled at later timepoints (compare Fig. 4F′ with Fig. 4L′). These findings indicate that timely SJ formation requires PAR-3 and PKC-3, but that some degree of PAR-3- and PKC-3-independent junction assembly occurs in later stages, and provide further evidence that the AJ and SJ proteins contribute to different structures.

### PAR-3 and PKC-3 contribute to apical exclusion of LET-413

In addition to its roles in junction positioning and maintenance, aPKC phosphorylates basolateral proteins, removing them from the apical domain in many epithelial tissues (Betschinger et al., 2003; Castiglioni et al., 2020; Ventura et al., 2020; Yamanaka et al., 2003). We therefore asked whether PKC-3 or PAR-3 is required during basolateral domain establishment in the embryonic intestine. LET-413 membrane localization was similar to controls in stage 3 PAR-3^gut(−)^ or PKC-3^gut(−)^ embryos (Fig. 5A-C). In stage 4 and 1.5-fold control embryos, LET-413 was excluded from the apical surface and localized basal to DLG-1 in junctions and to basolateral membranes (Fig. 5A′,A″,D) (Legouis et al., 2000). LET-413 was partially excluded from the apical surface of the intestine in stage 4 and 1.5-fold PKC-3^gut(−)^ embryos, although to a lesser degree than in control embryos (Fig. 5B′,B″,D). LET-413 was often absent from junctions in PKC-3^gut(−)^ 1.5-fold stage embryos but, where present, also localized basal to DLG-1 (Fig. 5E). Additionally, both junctional LET-413 and DLG-1 were separated by a smaller distance across the apical surface in PKC-3^gut(−)^ 1.5-fold embryos than in controls (Fig. 5E). Similar results were found for the exclusion of LGL-1 from the apical surface in PKC-3^gut(−)^ embryos (Fig. S7). LET-413 was also partially excluded from regions of the membrane in PAR-3^gut(−)^ embryos. These regions of LET-413 exclusion were initially flanked by DLG-1 puncta (stage 4, Fig. 5C′, arrowhead in inset) and eventually surrounded by DLG-1 rings (1.5-fold, Fig. 5C″,D). As there is no continuous apical surface in PAR-3^gut(−)^ embryos, we did not observe continuous exclusion of LET-413 along the intestinal midline. In both PAR-3^gut(−)^ and PKC-3^gut(−)^ embryos, the junctional LET-413 enrichment was reduced or absent (Fig. 5B″,C″). We were unable to analyze the localization of LGL-1 in PAR-3^gut(−)^ embryos, as we observed synthetic lethality in worms expressing both fluorescently tagged PAR-3 and LGL-1 (Fig. S7). Taken together, these results indicate that PAR-3 and PKC-3 are required for normal exclusion of LET-413 from the apical surface, and are required for the junctional, but not basolateral organization of LET-413. They also suggest that localization of PKC-3 by PAR-3 is not required for local basolateral domain establishment.

### Aspects of intestinal polarity arise locally but not globally upon PAR-3 depletion

**Fig. 6.**
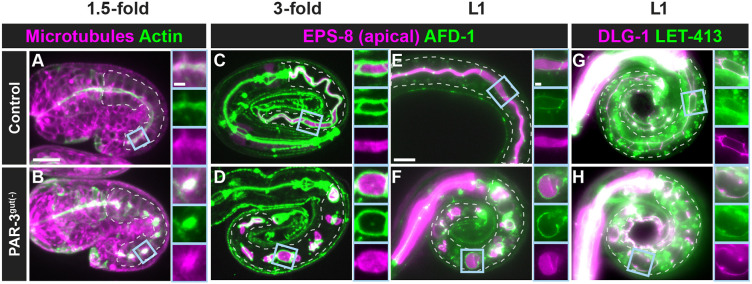
**Local apico-basolateral polarity is established in the absence of PAR-3.** (A,B) Localization of ACT-5 (actin, green) and TBA-1 (α-tubulin, magenta) transgenes in live 1.5-fold control (*n*=1) and PAR-3^gut(−)^ (*n*=10) embryos using confocal microscopy. (C-H) Localization of the indicated endogenously tagged proteins in fixed control (*n*=6) (C) and PAR-3^gut(−)^ (*n*=7) (D) 3-fold embryos using confocal microscopy and live control (E,G) and PAR-3^gut(−)^ (F,H) L1 larvae using a compound microscope. Magnified views of the boxed regions are shown on the right of each panel. Scale bars: 10 µm (panels); 2 µm (magnified views). Control (EPS-8;AFD-1/afadin, *n*=13; DLG-1;LET-413/Scribble, *n*=10); PAR-3^gut(−)^ (EPS-8;AFD-1/afadin, *n*=26; DLG-1;LET-413/Scribble, *n*=14).

The unexpected assembly of junctional rings in 1.5-fold PAR-3^gut(−)^ embryos raised the possibility that other aspects of apico-basolateral polarity could be established in the absence of PAR-3. To test this hypothesis, we examined apical, junctional, and basolateral structures in PAR-3^gut(−)^ 1.5-fold- to 3-fold-stage embryos and in newly hatched L1 larvae. In 1.5-fold control embryos, both tubulin and actin localized along the apical surface (Fig. 6A), consistent with the presence of apical microtubules, microvilli and the terminal web (Carberry et al., 2009; Feldman and Priess, 2012; Leung et al., 1999; MacQueen et al., 2005). In the intestines of 1.5-fold PAR-3^gut(−)^ embryos, we found discontinuous spheres of actin from which microtubules appeared to radiate (Fig. 6B) (Achilleos et al., 2010; Feldman and Priess, 2012). The actin-binding protein EPS-8, which localizes to the tips of microvilli (Croce et al., 2004), localized to the apical surface and was encircled by junctional AFD-1 in control 3-fold embryos and L1 larvae (Fig. 6C,E). In 3-fold PAR-3^gut(−)^ embryos and L1 larvae, AFD-1 also formed junctional rings that surrounded large spheres of EPS-8 (Fig. 6D,F). The spheres of EPS-8 and AFD-1 corresponded with the intestinal edemas observed in PAR-3^gut(−)^ L1 larvae in some places (Fig. 3C), but were also observed where edemas were not evident. Additionally, LET-413 appeared to be excluded from these apical-like regions, localizing with DLG-1 in PAR-3^gut(−)^ L1 larvae, similar to controls (Fig. 6G,H). Thus, small groups of cells eventually assembled apico-basolaterally polarized regions in the absence of PAR-3, suggesting an ability to establish some degree of local but not global tissue polarity.

To determine whether PAR-3 was also required for global polarity maintenance, we depleted PAR-3 from the intestine after global polarity was established, with PAR-3 depletion beginning at the 1.5- to 2-fold stage and completely removed by the 2.5-fold and later stages [PAR-3^lategut(−)^] (Fig. S8). Unlike PAR-3^gut(−)^ worms, PAR-3^lategut(−)^ worms grew at the same rate as controls (Fig. S8), indicating that PAR-3 is not required for polarity maintenance and consistent with the observation that PAR-3 is dispensable for polarity after the L1 larval stage (Castiglioni et al., 2020).

### HMR-1 is required for local polarity establishment in the absence of PAR-3

**Fig. 7.**
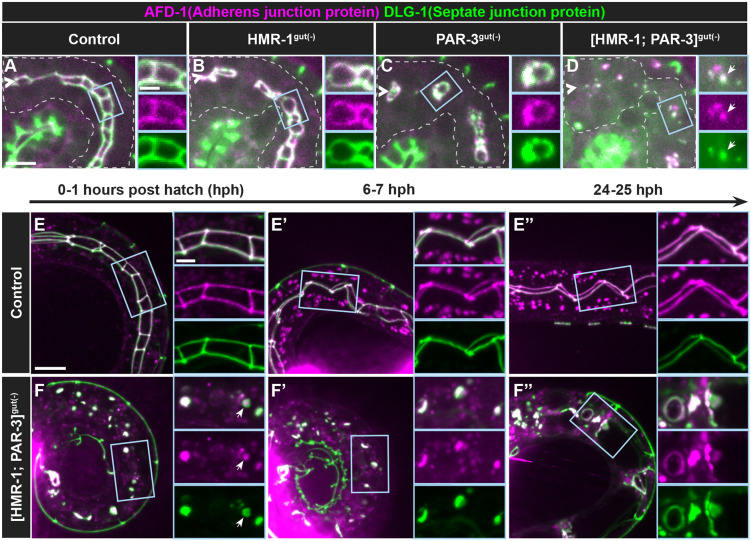
**HMR-1 is required for local polarity establishment in the absence of PAR-3.** Live images of endogenously tagged AFD-1 (magenta) and DLG-1 (green) localization. (A-D) Dorsolateral images of 1.5-fold intestines from control (*n*=4), HMR-1^gut(−)^ (*n*=9), PAR-3^gut(−)^ (*n*=5) and (HMR-1;PAR-3)^gut(−)^ (*n*=5) embryos. Magnified views of the boxed regions are shown on the right of each panel. (E-F″) Time courses in control or (HMR-1;PAR-3)^gut(−)^ L1 larvae for hours post hatching (hph). Magnified views of the boxed regions are shown on the right of each panel. Control (0-1 hph, *n*=17; 6-7 hph, *n*=13; 24-25 hph, *n*=9); (HMR-1;PAR-3)^gut(−)^ (0-1 hph, *n*=17; 6-7 hph, *n*=13; 24-25 hph, *n*=9). Scale bar: 5 µm (panels in A-D); 10 µm (panels in E-F″); 2 µm (magnified views in A-F″).

As junctional rings formed following PAR-3 depletion, we hypothesized that the junctions themselves could act redundantly to establish local cell polarity. We first tested whether HMR-1 is required for the assembly of junctions in the intestine. Both AFD-1 and DLG-1 localized in 1.5-fold HMR-1^gut(−)^ embryos (Fig. 7B), albeit into more broken structures than the ladder-like junctions in controls (Fig. 7A,B; Fig. S9). These structures were more continuous in 1.5-fold HMR-1^gut(−)^ embryos than in 1.5-fold PAR-3^gut(−)^ embryos, in which occasional DLG-1 structures formed at midline vertices (Fig. 7C; Fig. S9). We next co-depleted HMR-1 and PAR-3 in the intestine [(HMR-1;PAR-3)^gut(−)^] to test whether HMR-1 is required for the organization of junctions in the absence of PAR-3. We found that junctional rings were absent in the intestines of 1.5-fold (HMR-1;PAR-3)^gut(−)^ embryos (Fig. 7D). AFD-1 and DLG-1 localized to small puncta on cell membranes throughout the intestine (Fig. S9). To better understand local polarity in (HMR-1;PAR-3)^gut(−)^ larvae, we followed individual L1 animals over time. In newly hatched [0-1 h post hatching (hph)] control L1 and older larvae, AFD-1 and DLG-1 localized into continuous junctions (Fig. 7E-E″). In (HMR-1;PAR-3)^gut(−)^ L1 larvae, AFD-1 and DLG-1 appeared to be largely colocalized in puncta (Fig. 7F). We occasionally observed a hollowing of DLG-1 away from the center of the puncta. By 6 hph, the same L1 larvae showed an expansion of edemas throughout the intestine, but AFD-1 and DLG-1 remained largely colocalized in puncta and sometimes in small rings that did not surround the detectable edemas (Fig. 7F′). Surprisingly, as these L1s aged (24-32 hph), AFD-1 and DLG-1 often formed rings that partially or completely enclosed edemas (Fig. 7F″). Edemas appeared prior to the formation of junctional rings, suggesting that these rings may arise stochastically through limited space and protein-protein interactions. Although we cannot entirely rule out the possibility that small amounts of PAR-3 or HMR-1 were present in (HMR-1;PAR-3)^gut(−)^ larvae, the asymmetric organization of AFD-1 and DLG-1 in rings suggests that aspects of local polarity may still arise in the absence of both PAR-3 and HMR-1, and highlights the robustness and redundancy of polarization mechanisms.

## DISCUSSION

Through the live analysis of polarization and tissue specific depletion in the *C. elegans* embryonic intestine, we establish a paradigm wherein epithelial polarity at the local and global levels are temporally and genetically separable. Local polarity is initiated prior to global polarity before the apical surfaces of all cells align along the intestinal midline. PAR-3 is required for global polarity establishment across the tissue but is dispensable for the establishment of local pockets of polarity, which, although delayed, can ultimately still form through HMR-1-dependent mechanisms. Our results indicate that local polarity establishment is robust but insufficient to establish global apico-basolateral polarity without additional tissue-level coordination by PAR-3.

### Local versus global polarity establishment

We found *in vivo* evidence that apico-basolateral polarity is established at two separable levels: (1) locally – individual epithelial cells define an apico-basolateral axis; and (2) globally – apico-basolateral axes are coordinately aligned, thereby creating a continuous apical surface across the tissue. The anatomy of the *C. elegans* intestine allowed us to visualize the natural temporal separation between local and global polarization. Unlike Madin–Darby canine kidney (MDCK) cysts in which cell division across the future apical surface guides local and global apical establishment via the remnant midbody (Li et al., 2014; Lujan et al., 2017), precursors to the E16-stage intestinal primordium divide parallel to, instead of across, the future apical surface, requiring a 90° rotation of structures such as the centrosomes and the attached nucleus to arrive at the apical surface (Feldman and Priess, 2012; Leung et al., 1999). Additionally, apical proteins are initially positioned as lateral puncta on the membranes of adjacent non-sister cells at E16 and must move to the future apical surface. The asymmetric localization of lateral puncta suggests that local polarization events begin well before global polarity establishment. However, basolateral proteins were not excluded from either the lateral puncta or the nascent apical surface, indicating that although local polarization is an early process, the definition of a basolateral surface normally appears concomitant with global polarity establishment. The separation of local and global polarity may be common among epithelia as the mammalian PAR-3 ortholog similarly localizes to asymmetric patches on lateral membranes prior to becoming globally oriented in the polarizing mouse kidney nephron (Yang et al., 2013).

Local and global polarity establishment are also genetically separable. Degradation of PAR-3 initially impeded local polarity as AJ proteins did not form lateral puncta and instead localized weakly along lateral cell membranes, indicating that PAR-3 is required for the correct timing of local polarity establishment. However, some aspects of local polarity were established at later timepoints when HMR-1-dependent junctional rings surrounded proteins associated with the apical cytoskeleton and partially excluded basolateral proteins. These locally polarized regions were insufficient to establish global polarity. As global polarity establishment may be dependent upon the precise developmental timing of local polarity establishment, the delay in local polarity establishment likely contributes to the absence of global polarity in PAR-3^gut(−)^ embryos.

Separation of local and global polarity establishment may highlight different protein requirements in the evolution of multicellularity and explain the robustness of local polarity establishment. In single-cell contexts, local polarity establishment is robust. For example, in *Saccharomyces cerevisiae*, stochastic changes in protein concentrations at the plasma membrane are sufficient to drive polarity establishment in the absence of other cues (Chiou et al., 2017; Wu and Lew, 2013). In the one-cell *C. elegans* zygote, several redundant processes ensure the robustness of anterior-posterior polarity establishment such that the loss of individual polarity proteins (e.g. PAR-2, ECT-2 or LGL-1) or processes (e.g. actomyosin contractility) does not prevent polarization (Hoege et al., 2010; Motegi and Seydoux, 2013; Motegi et al., 2011; Zonies et al., 2010). However, coordinate polarity orientation of multiple individual cells is less likely to occur correctly through stochastic processes; thus, global polarity establishment may be more prone to disruption without additional reinforcing mechanisms.

The mechanisms required for local polarization of lateral puncta are unknown. Unlike in MDCK cysts, midbodies are unlikely to provide a polarizing cue as they are positioned on opposite membranes from lateral puncta (Bai et al., 2020; Li et al., 2014; Lujan et al., 2017). Instead, mitosis within the intestinal primordium may promote the formation of lateral puncta by trafficking polarity proteins along astral microtubules to their lateral attachment sites or by increasing the concavity of the cell membrane, which can promote local accumulation of polarity proteins (Chiou et al., 2017; Feldman and Priess, 2012). Alternatively, as transmembrane proteins at yeast bud scars provide polarizing cues, earlier intestinal cell divisions may locally enrich transmembrane proteins like cadherins, nectins or claudins at lateral membranes, which could then recruit apical and junctional proteins (Chiou et al., 2017). Recently, PAR-3 and other apical and junctional proteins have been found to undergo liquid-liquid phase separation, which is sufficient to promote puncta formation (Liu et al., 2020; Rouaud et al., 2020). Thus, interaction of PAR-3 with transmembrane and other polarity proteins, phase separation and simple reaction-diffusion may contribute to the establishment of local polarity puncta (Chiou et al., 2017; Wu et al., 2018).

The mechanisms that move lateral puncta, midbodies and basolateral proteins are also unknown, but the movement of all of these structures toward the midline is consistent with a global polarization event (Achilleos et al., 2010; Bai et al., 2020; Feldman and Priess, 2012). Actomyosin contractility produces cortical anterior flow during anterior-posterior polarity establishment in the one-cell *C. elegans* zygote and might be expected to similarly generate apical flow in the intestine (Goehring et al., 2011; Munro et al., 2004). However, acute actin disruption does not disrupt lateral puncta movement, suggesting that actomyosin contractility is not required for global polarization (Feldman and Priess, 2012). By contrast, disruption of microtubules strongly delayed the movement of lateral puncta, suggesting that microtubules are at least partially required for global polarization (Feldman and Priess, 2012).

### PAR-3 and PKC-3 have separable roles in polarity establishment and maintenance

PAR-3/Par3, PAR-6/Par6, and the kinase PKC-3/aPKC compose the apical PAR complex that is crucial for establishing apico-basolateral polarity in many epithelial tissues (Harris and Peifer, 2004, 2005; Hutterer et al., 2004; Totong et al., 2007; Von Stetina and Mango, 2015). Although the depletion of PAR-3, PAR-6 or PKC-3 from the embryonic intestine resulted in similar L1 larval phenotypes, including intestinal obstruction, the absence of a continuous lumen, edema formation and 100% larval arrest (this study; Sallee et al., 2021), we found that these terminal phenotypes arise for distinct reasons. Although we cannot rule out the possibility that our depletion efficiency varied between proteins, the differences between the PKC-3 and PAR-3 phenotypes are consistent with previous observations (Achilleos et al., 2010; Sallee et al., 2021; Totong et al., 2007).

We found that PKC-3 is not required for polarity establishment but for the remodeling and maintenance of global polarity like its binding partner PAR-6 (this study; Sallee et al., 2021). PKC-3 and PAR-6 localize interdependently and depletion of either protein results in gaps in the apical surface and AJ proteins, decreased exclusion of basolateral proteins from the apical surface, and discontinuous SJ protein localization (this study; Montoyo-Rosario et al., 2020; Sallee et al., 2021; Totong et al., 2007). These defects may arise during morphogenesis because of a failure to remodel apical junctions, the apical surface, or both. The SJ proteins DLG-1 and PAR-1 appear fragmented both in intestines and in seam cells depleted of PKC-3 (this study; Castiglioni et al., 2020), suggesting that PKC-3 may be broadly required to organize SJ proteins across epithelial tissues.

In contrast to PKC-3^gut(−)^ embryos, we found a severe delay in local polarization and complete disruption of global polarity establishment following PAR-3 depletion. PAR-3 is a scaffolding protein with an N-terminal oligomerization domain and PDZ domains required for formation of multimeric PAR-3/PAR-6/PKC-3 complexes (Chen and Zhang, 2013; Dickinson et al., 2017). Thus, depletion of PAR-3 could result in mislocalized PKC-3 activity, producing a very different phenotype than that of PKC-3 depletion. Alternatively, the PDZ and C-terminal domains of PAR-3 interact with multiple junctional proteins and membrane lipids, which may allow PAR-3 to position apical and junctional proteins independently of its interaction with PKC-3 (Chen and Zhang, 2013). Additionally, PAR-3 may regulate additional kinases such as the Pak1 orthologs MAX-2 or PAK-1 to establish polarity, as in the fly follicular epithelium and cultured mammalian intestinal cells (Aguilar-Aragon et al., 2018). Thus, PAR-3 may coordinate global polarity by positioning apical and junctional proteins as it spreads along the intestinal midline, explaining the more severe defects of PAR-3 depletion than PKC-3 depletion (this study; Achilleos et al., 2010).

### Rules that govern polarity establishment: when is PAR-3 required?

PAR-3 plays an essential role in polarity establishment in certain epithelia but is unnecessary in others, raising the question of in what contexts is PAR-3 necessary for polarity establishment (this study; Achilleos et al., 2010; Chen et al., 2018; Harris and Peifer, 2004; Shahab et al., 2015; Yang et al., 2013). Notably, PAR-3 only appears to be required during global polarity establishment in the embryonic *C. elegans* intestine as it is dispensable for polarity maintenance and intestinal function in larval and adult worms (this study; Castiglioni et al., 2020). We speculate that the differential requirements for PAR-3 across developmental times and tissues stems from differences in the asymmetric information available to cells prior to polarity establishment. PAR-3 may be necessary when polarity domains are established for the first time, but not when cells inherit asymmetric information from precursors or are born into already polarized tissues. For example, asymmetries between cell-contacting and contact-free surfaces are sufficient to drive PAR-3-independent radial polarization in early *C. elegans* embryos (Nance, 2014). PAR-3 is also dispensable for polarization in the *C. elegans* epidermis, which has an asymmetric contact-free surface that might coordinate global polarity (Achilleos et al., 2010). The *Drosophila* follicular epithelium and adult midgut do not require PAR-3 and experience asymmetries at future apical and basal surfaces through asymmetric contact with the germline and/or basal ECM, which may provide both physical and chemical cues to coordinate global polarity (Chen et al., 2018; Schneider et al., 2006; Shahab et al., 2015; Tanentzapf et al., 2000). Thus, extrinsic informational cues such as chemical signaling gradients, contact with the ECM or the presence of external contact-free surfaces might guide global polarization independently of PAR-3 (Pickett et al., 2019). The polarizing intestine does not have a contact-free surface, does not make heterotypic contacts at the future apical surface and does not contact the basal ECM, perhaps necessitating PAR-3 for global coordination of polarity establishment in the absence of other cues (Nance et al., 2003; Nance and Priess, 2002; Rasmussen et al., 2013). This role is likely in concert with other developmental information, as regions of local polarity in PAR-3^gut(−)^ embryos appeared more centrally localized than would be expected by chance. Indeed, PAR-3 functions together with other asymmetric information in both the *Drosophila* blastoderm and the one-cell *C. elegans* zygote when polarity domains are initially established (Dickinson et al., 2017; Harris and Peifer, 2004; Kemphues et al., 1988; Motegi et al., 2011).

The results presented here demonstrate the power of holistic *in vivo* studies, in which polarizing tissues encounter their normal external cues, to understand the many mechanisms that drive local and global polarity establishment in different epithelia. Additional systematic studies of polarity pathways are necessary to understand when and how different proteins contribute to the coordination of local and global polarity establishment. Such studies will undoubtedly provide insight into the evolution of multicellularity as well as prove informative to how changes in polarity protein expression contribute differentially to a multitude of epithelial diseases.

## MATERIALS AND METHODS

### *C. elegans* strains and maintenance

*C. elegans* strains were maintained on nematode growth medium (NGM) plates coated with a lawn of *Escherichiacoli* OP50 bacteria at 15-20°C as previously described (Sulston and Brenner, 1974). Embryos and larvae used for experiments were collected from 1- to 2-day-old adults, with the exception of PAR-3^gut(−)^ and (PAR-3;HMR-1)^gut(−)^ experiments in which 3-day-old adults were occasionally used owing to the difficulty in obtaining balancer (−) adults. A full list of strains used in this study is available in Table S1.

### CRISPR cloning and editing

Wormbase was used to identify all known isoforms of targeted genes (Davis et al., 2022) and the UCSC Genome Browser (https://genome.ucsc.edu/) *C. elegans* assembly October 2010 (WS220/ce10) was used to design short guide RNAs (sgRNAs). New CRISPR alleles were generated using the self-excision cassette (SEC) method as previously described (Dickinson et al., 2015). The pDD162 plasmid (Addgene #47549) was used to deliver Cas9 and sgRNAs modified using the Q5 site-directed mutagenesis kit (New England Biolabs) for sequence-specific CRISPR editing of each gene. Homology-guided repair templates were generated by Phusion High-Fidelity DNA polymerase (Thermo Fisher Scientific)-mediated PCR amplification of appropriate homology arm sequences for N-terminal, C-terminal or internal-fluorophore tags. Homology arms were then cloned into an SEC backbone plasmid [pJF250 (Sallee et al., 2018), pDD282 (Addgene #66823) or pLC019 (this paper, L.C.)] with Gibson assembly (NEBuilder HiFi DNA assembly master mix, New England Biolabs). A mixture of the modified SEC repair plasmid (25 ng/µl) and modified Cas9/sgRNA plasmid (50 ng/µl) was injected into both gonad arms of 1-day-old *zif-1(gk117)* adult hermaphrodites. Injected worms were recovered and treated as previously described to isolate new CRISPR edits and excision of the SEC (Dickinson et al., 2015). Alleles were tagged with ZF::GFP::3×FLAG, GFP::3×FLAG or mScarlet::3×FLAG. New CRISPR alleles were backcrossed twice with *zif-1(gk117)* worms prior to use in experiments. New worm lines generated through CRISPR editing are listed in Table S1. All sgRNA and homology arm sequences, plasmids and primers used to generate new CRISPR alleles are available in Table S2.

### Obtaining gut(−) embryos and L1 larvae

All intestine-specific depletion strains were maintained with the ZF::GFP allele over an appropriate balancer (see Table S1), with the exception of LGL-1::ZF::mScarlet, which was maintained unbalanced. To obtain gut(−) embryos and L1 larvae, L4 and young adult hermaphrodites lacking the balancer were picked to a fresh plate and maintained at 20°C overnight. The following day, the balancer (−) adults were transferred to 30 µl M9 on a teflon coated slide, washed three times with M9, and incubated in a humidity chamber for 2.5-3 h at room temperature to obtain gut(−) embryos of the appropriate stages. Worms were cut open to release the gut(−) embryos, which were then imaged (see below) and scored for defects. To obtain gut(−) L1s, the young balancer (−) adults were transferred to a fresh small NGM plate and allowed to lay eggs at 20°C for 1-4 h. Adults were then removed and plates returned to 20°C for 12-24 h when L1s were scored for defects. This strategy depletes both maternal and zygotic proteins from the intestine, as both supplies of the protein is ZF tagged and subject to degradation. Depletion was verified by loss of GFP fluorescence at the intestinal midline (Fig. S4). While the ZF degradation system does not create a null line, it has been used to robustly deplete ZF-tagged proteins (Abrams and Nance, 2021; Liang et al., 2020; Magescas et al., 2021; Sallee et al., 2021, 2018; Sanchez et al., 2021) and we see strong depletion.

### Immunofluorescence

One- to 2-day old hermaphrodites were incubated in 30 µl M9 buffer in a humidity chamber at room temperature for 2.5-3.5 h, and were then cut open to release their embryos which were stained as previously described (Leung et al., 1999). Embryos were attached to a poly-L-lysine-coated slide with Teflon spacers and rapidly frozen on dry ice. Embryos were permeabilized with the freeze-crack method and fixed in −20°C 100% methanol for 5 min. Slides were then washed twice in 1× PBS for 5 min at room temperature, followed by a single 5-min wash in 1× PBS with 0.1% Tween 20 (PBT). Embryos were incubated in primary antibody solutions in a humidity chamber at 4°C overnight. Slides were washed three times in PBT for 5 min each, then incubated in secondary antibody solutions in a humidity chamber at 37°C for 1 h. Slides were then washed twice in PBT for 5 min each, followed by a 5-min wash in 1× PBS at room temperature. Vectashield (Vector Laboratories) was added to each slide and coverslips sealed to slides with fingernail polish. Slides were imaged on a Nikon Ti-E inverted spinning disk confocal microscope (see below). The following primary antibodies were used: anti-GFP (Abcam, AB6556, 1:200) and anti-PAR-3 (Developmental Studies Hybridoma Bank, P4A1, 1:25). The following secondary antibodies were used: Cy3 anti-mouse IgG (Jackson ImmunoResearch, 115165166, 1:200), Alexa Fluor 488 goat anti-rabbit IgG (Jackson ImmunoResearch, 111545144, 1:200), Alexa Fluor 488 goat anti-mouse IgG (Jackson ImmunoResearch, 115545166, 1:200), Alexa Fluor 647 donkey anti-rabbit IgG (Jackson ImmunoResearch, 711605152, 1:50). DAPI (Sigma-Aldrich, D9542, 1:10,000) staining was used to visualize nuclei.

### Microscopy

Embryos were collected from 1- to 2-day-old hermaphrodites, incubated in M9 buffer for 2.5-3.5 h at room temperature and used for live imaging. For (PAR-3;HMR-1)^gut(−)^, embryos were picked from small plates containing adult hermaphrodites that lacked the hT2 balancer. Embryos were imaged live and mounted on 3% agarose pads in 1× M9. Images were acquired on a Nikon Ti-E inverted microscope (Nikon Instruments, Melville, NY, USA) with a 60× Oil Plan Apochromate (NA=1.4), an Andor Ixon Ultra back thinned EM-CCD camera, using 405 nm, 488 nm and/or 561 nm lasers, and a Yokogawa X1 confocal spinning-disk head objective controlled by NIS Elements software (Nikon). Images were acquired at a *z*-sampling rate of 0.3-0.5 µm. Some L1 larvae were imaged using a Nikon Ni-E compound microscope with a 100× Oil Plan Apochromat (NA=1.45) objective. Maximum-intensity projections were made with FIJI. Imaris was used for three-dimensional rotation. FIJI, Imaris and Photoshop were used for time-lapse movies.

### Embryo staging

Control embryos were separated into stages 1, 2, 3 or 4 based on the morphology of the embryo and the localization of PAR-3 or PKC-3 in the intestine and pharynx. Stage 1 embryos were identified if the overall shape was relatively oval and by the localization of PAR-3 or PKC-3 into puncta at the lateral cell membranes of non-sister intestinal cells. Stage 2 embryos were identified if they had a similar oval shape, but the PAR-3 or PKC-3 puncta were observed at the intestinal midline instead of the lateral membranes. Stage 3 embryos were identified by their slightly pinched ‘bean’ shape with the anterior part of the embryo slightly wider than the posterior part of the embryo, and PAR-3 or PKC-3 localized in a single continuous line from the pharynx through the intestine. Stage 4 embryos had a classic ‘comma’ shape and PAR-3 or PKC-3 localized in a single continuous line from the pharynx through the intestine. For PAR-3^gut(−)^ or PKC-3^gut(−)^ embryos, stage 3 was identified by the shape of the overall embryo and the apical localization of PAR-3 or PKC-3 in a single continuous line within the pharynx.

### Image analysis and statistical analysis

#### Quantification of midline enrichment and basolateral exclusion

To determine whether basolateral proteins were enriched at the midline and when they became excluded from this surface, we analyzed images from live embryos at stages 1, 2, 2.5/3 and 4. Using FIJI, we used endogenous PAR-3::mCherry or *end-1*p-driven BFP::CAAX signal to select the brightest plane to define the intestinal midline and made a sum *z*-projection of three slices around this plane for further analysis. Boxes that were 2-µm wide were drawn by hand at the apical and lateral surfaces to define regions of interest (ROIs) for basolateral proteins. Apical enrichment was determined by dividing the average apical signal by the average lateral signal for each embryo. A 1-µm-wide line segment was drawn across the midline at Int2 and pixels within 5 µm of the midline were selected to generate a line profile. Plots of apical enrichment and profile plots were generated and normalized in R with ggplot2.

To determine whether basolateral proteins were excluded from the apical surfaces of intestines of PAR-3^gut(−)^ or PKC-3^gut(−)^ 1.5-fold embryos, we used FIJI to make sum *z*-projections of five slices using DLG-1:mScarlet to define the apical surfaces for LET-413, and sum *z*-projections of three slices using lifeAct:RFP to define the apical surfaces for LGL-1. Boxes that were 1-µm wide were drawn by hand in the posterior region of the intestine (between Int5-Int8) at the apical and lateral surfaces to define ROIs for basolateral proteins and three 1-µm-wide boxes were drawn to measure the cytoplasmic signal. The corrected signal intensity was determined by subtracting the cytoplasmic signal from the apical and lateral signals. Plots and two-tailed unpaired Student's *t*-tests were run with GraphPad to test for significance. The partial exclusion of LET-413 from the intestinal midline of PKC-3^gut(−)^ worms was similar to the partial exclusion of LGL-1 from the midline in PAR-6^gut(−)^ embryos, but differed from the higher levels of LET-413 detected at the midline of PAR-6^gut(−)^ worms that we previously reported (Sallee et al., 2021). These differences are likely due to measurements made in the anterior rather than more posterior regions of the intestine and may also reflect differences between the LET-413 transgene used in our previous study as opposed to the endogenously tagged LET-413 used here (Sallee et al., 2021). To investigate the reasons for these differences further, we also analyzed LGL-1 in the anterior intestine of PKC-3^gut(−)^ and control embryos using the same analysis that we used previously. Briefly, a single *z*-slice was taken and the anterior intestinal cells (Int1 and Int2) were analyzed by drawing 1-µm-wide boxes at the apical and lateral surfaces to define ROIs for the anterior LGL-1 signal, and three 1-µm-wide boxes were drawn to measure the cytoplasmic signal. Overall, these two different methods of analysis provided similar results, with higher LGL-1 signal detected at the apical surfaces in both the anterior and posterior part of the intestine of PKC-3^gut(−)^ embryos compared with controls (Fig. S7). However, we did not detect a difference between the apical and lateral LGL-1 signals in the anterior part of the intestine and, instead, detected a significant difference between the lateral signals in control and PKC-3^gut(−)^ embryos, unlike in the posterior part of the intestine, indicating slight differences between these two methods of analysis (Fig. S7). The similarities we found with either method of analysis suggest that PAR-6 and PKC-3 play a similar role in completely excluding basolateral proteins from the apical surface.

#### Quantification of the distance between junctional intensity peaks

We used FIJI to determine the distance between the left and right sides of the junctions across the midline. For comparisons of AJ proteins and SJ proteins, we analyzed 1.5-fold embryos expressing two endogenously tagged junctional proteins, each tagged with a different fluorophore. A sum *z*-projection of five slices was made around the brightest intestinal plane based on the GFP or mNeonGreen junctional signal. Three 1-µm-wide line segments were drawn across the midline between Int5 and Int8 to generate line profiles for both junctional channels. Measurements were made near the middle of each intestinal ring in order to accurately measure the distance between the left and right sides of the junction, while avoiding the junctional signal between anterior-posterior neighbors. The difference between the two brightest peaks (highest signal intensity) for each line profile was determined to find the distance between left and right sides. The distances were averaged from the three profile lines for each embryo. GraphPad was used to conduct paired two-tailed *t*-tests to determine whether there were statistically significant differences in the distances between the paired junctional components (HMR-1 versus DLG-1; HMR-1 versus AFD-1; AFD-1 versus DLG-1; AFD-1 versus PAR-1; and DLG-1 versus PAR-1).

### L1 arrest and larval growth assays

To assess embryonic lethality and larval growth, ten L4 hermaphrodites lacking the balancer and 30 control L4 hermaphrodites [ten with the ZF::GFP tag alone, ten *zif-1(gk117)* and ten *zif-1(gk117);*(intestine-specific ZIF-1)] were picked to fresh plates and maintained at 20°C overnight. The following day, 1-day-old adults were singled onto ten plates and allowed to lay embryos for 4 h at 20°C. Adult worms were then removed and embryos were counted at least twice. Plates were returned to 20°C for 24 h. Embryos were counted again and scored as unhatched or dead. The difference between embryos counted on the first day and embryos counted 24 h later was used to determine the number of L1 larvae on each plate. Plates were returned to 20°C for 24 h. Embryos, L2 larvae and L3 larvae were counted at least twice and then plates were returned to 20°C for 24 h. On the final day (72 h after adult hermaphrodites were singled), all larval stages (L1 to adult) were counted. After 72 h, most L1 larvae were still alive but appeared sick, only moving upon touch stimulation.

### L1 Smurf feeding assays

One- to 2-day-old adult hermaphrodites were picked to fresh, small NGM plates and allowed to lay embryos for up to 16 h at 20°C. Hatched L1 larvae were picked into a 30 µl drop of standard overnight OP50 bacterial culture and 10 µl of 20% blue food coloring (FD&C Blue #1 Powder, Flavors and Color, acquired through Amazon) in water was added to the bacteria. L1 larvae were incubated with the blue-dyed OP50 bacteria in a humidity chamber at room temperature for 3 h. To collect larvae, the dyed OP50 solution was transferred and spread over a medium NGM plate. L1 larvae were located using oblique illumination and were then mounted in 2 mM levamisole on a 3% agarose pad and imaged on a Nikon Ni-E compound microscope with a 100× Oil Plan Apochromat (NA=1.45) objective and an additional (2×) digital zoom with a Google Pixel 4, mounted to the microscope eyepiece. Rarely, an L1 larva was damaged in the process of transferring it to levamisole. These larvae appeared desiccated, and their entire bodies were dyed blue. Damaged L1 larvae were excluded from analysis.

## Supplementary Material

10.1242/develop.200325_sup1 Supplementary information
