# Supplementary material for: Separable mechanisms drive local and global polarity establishment in the Caenorhabditis elegans intestinal epithelium
Source: Development. 2022 Nov 16;149(22):dev200325. doi: 10.1242/dev.200325 (PMC9845746; doi:10.1242/dev.200325)
Supplement: Supplementary information [file develop-149-200325-s1.pdf]

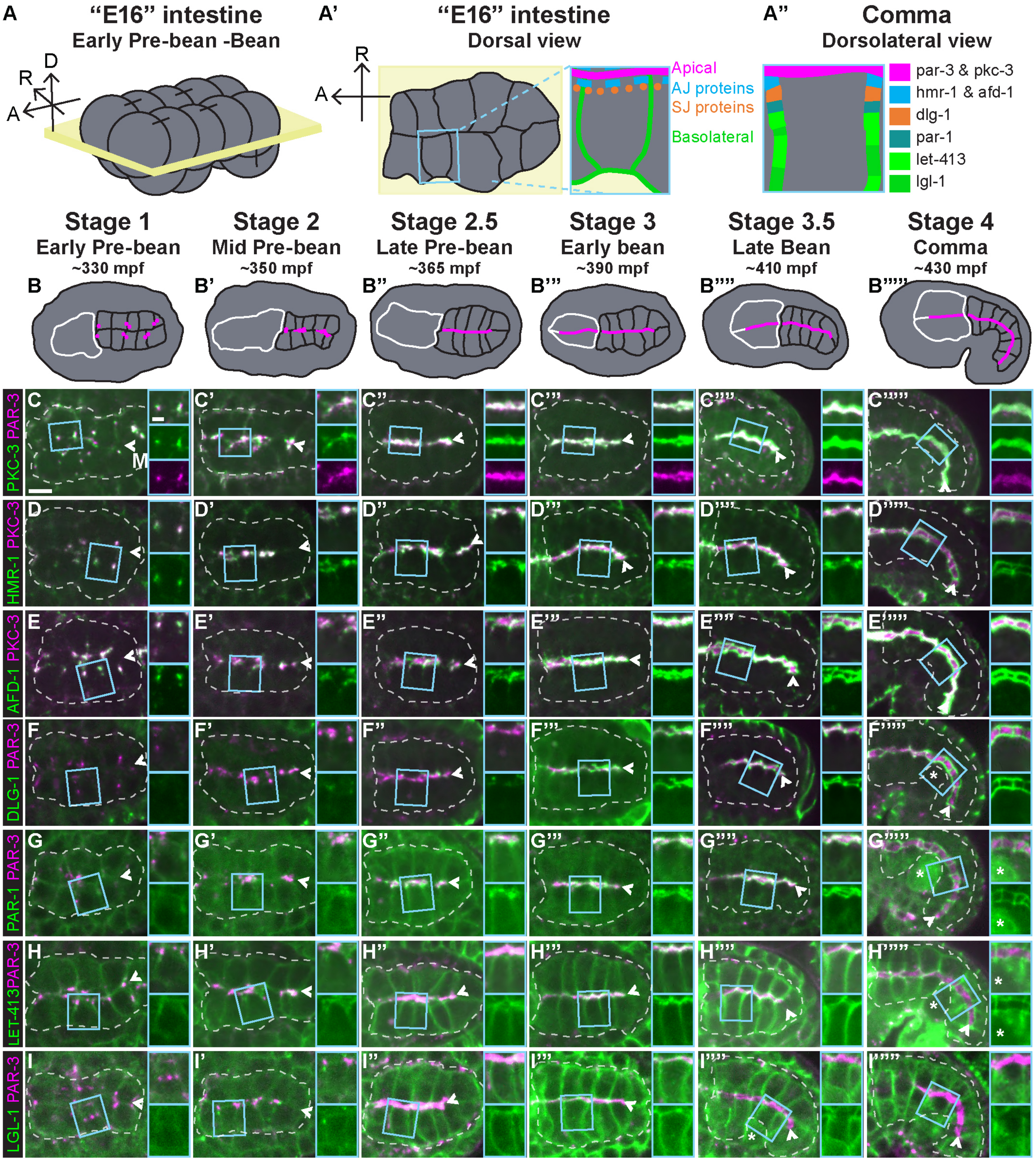

Development • Supplementary information

Fig. S1. Apical, junctional, and basolateral proteins localize dynamically during global polarity establishment. A) Schematic lateral 3D view of the E16 intestine and the imaging plane (yellow). A') Dorsal view of the E16 intestine, showing the imaging plane (yellow) and the relative position of apical, AJ, SJ, and Basolateral proteins as indicated. A''') Final organization of indicated polarity proteins in the Stage 4 intestine. B-B''''') Schematics of *C. elegans* embryonic morphogenesis from early pre-bean (Stage 1) to comma (Stage 4), with minutes post-fertilization (mpf) indicated. Intestinal membranes in black lines, intestines marked with white boxes, and pharynx outlined in white. C-I''''') Dorsal view of co-localization of endogenously tagged indicated proteins (green) and PAR-3 or PKC-3 (magenta) in Stage 1 (PKC-3, n=12; HMR-1, n=11; AFD-1, n=8; DLG-1, n=14; PAR-1, n=10; LET-413, n=11;LGL-1, n=12), Stage 2 (PKC-3, n=11; HMR-1, n=13; AFD-1, n=26; DLG-1, n=13; PAR-1, n=12; LET-413, n=8;LGL-1, n=9), Stage 2.5 (PKC-3, n=4; HMR-1, n=8; AFD-1, n=5; DLG-1, n=17; PAR-1, n=13; LET-413, n=13;LGL-1, n=11), Stage 3 (PKC-3, n=5; HMR-1, n=11; AFD-1, n=8; DLG-1, n=17; PAR-1, n=11; LET-413, n=13;LGL-1, n=9), Stage 3.5 (PKC-3, n=3; HMR-1, n=1; AFD-1, n=7; DLG-1, n=12; PAR-1, n=12; LET-413, n=13;LGL-1, n=7), or Stage 4 (PKC-3, n=16; HMR-1, n=5; AFD-1, n=9; DLG-1, n=12; PAR-1, n=12; LET-413, n=15;LGL-1, n=12) intestines. All images are maximum intensity projections from live imaging. Intestines outlined by white dashed lines, midlines indicated by arrowheads. Enlarged versions of boxed regions shown to right. Scale bar = 5 μm for panels and 2 μm for boxed insets. Asterisks mark germ cells. I-I''')

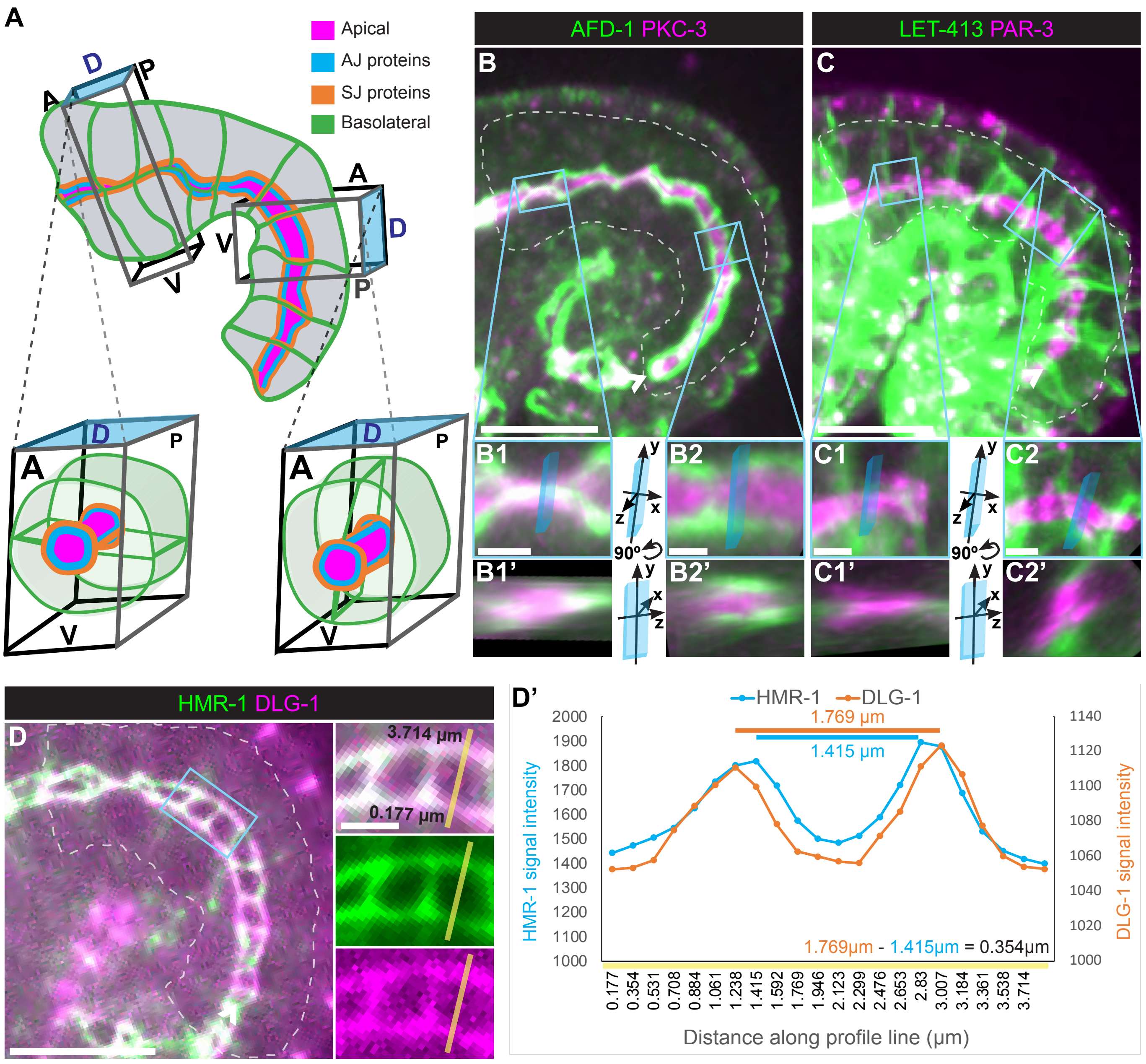

Development • Supplementary information

Fig. S2. Junctional organization along the apical-basolateral axis. A) Schematic of apical (magenta), AJ-proteins (blue), SJ-proteins (orange), and basolateral proteins (green) in dorsolateral view of an embryonic 1.5-fold stage intestine. Boxes around intestinal ring (Int)2 and Int7. Boxes rotated as indicated to show *en face* view of Int2 and Int7, depicting organization of AJ-, SJ-, and basolateral proteins relative to the apical surface. B-C) Lateral view of endogenously tagged indicated proteins in 1.5-fold stage embryonic intestines. B1-C2) Enlarged versions of boxed regions from B and C showing Int2 and Int7. B1'-C2') Regions indicated by blue planes in B1-C2 rotated 90º to show the *en face* view of apical, junctional, and basolateral protein organization in Int2 and Int7. D) Lateral view of HMR-1 (green) and DLG-1 (magenta) localization in 1.5-fold stage embryonic intestine. Enlarged version of boxed region shown to right, with example of a profile line used to measure the signal intensity of junctional proteins and distance between the left and right sides of the intestine shown in yellow. D') Example of a profile plot showing the signal intensity for HMR-1(blue) and DLG-1 (orange). Distance between the peaks was used to determine the distance between the left and right sides of the junctions. These were calculated for each pair of proteins, and the difference taken to determine the relative localization of proteins within the junctions. Scale bar = 10 µm for A,B,&C, 2 µm for boxed insets, and 1 µm for B2.

Development • Supplementary information

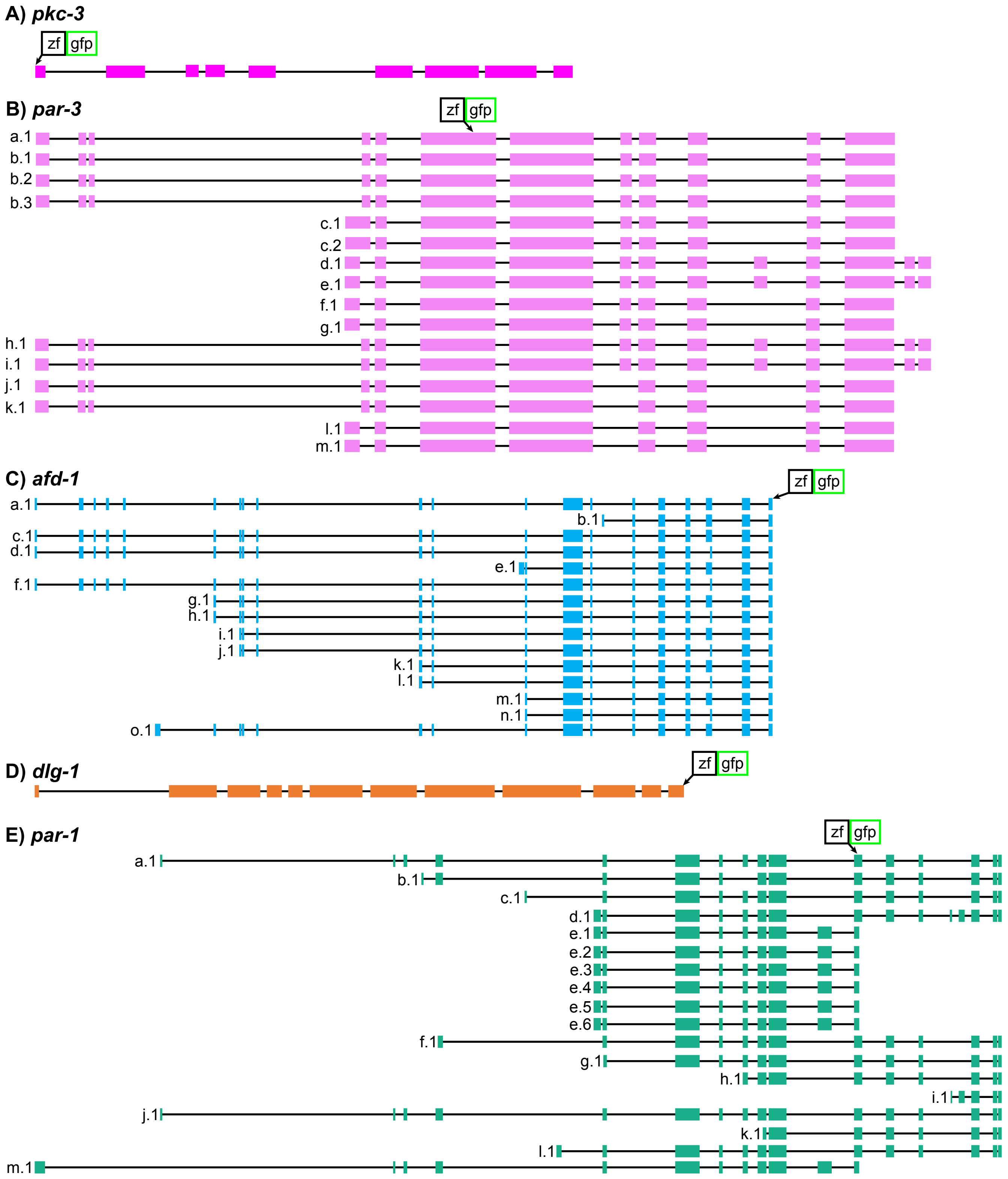

Fig. S3. Location of *zf:gfp* tags for endogenously tagged CRISPR strains for the following genes A) *pkc-3* B) *par-3* C) *afd-1*, D) *dlg-1,* and E) *par-1.* Gene maps from WormBase (Davis et al 2022).

Development • Supplementary information

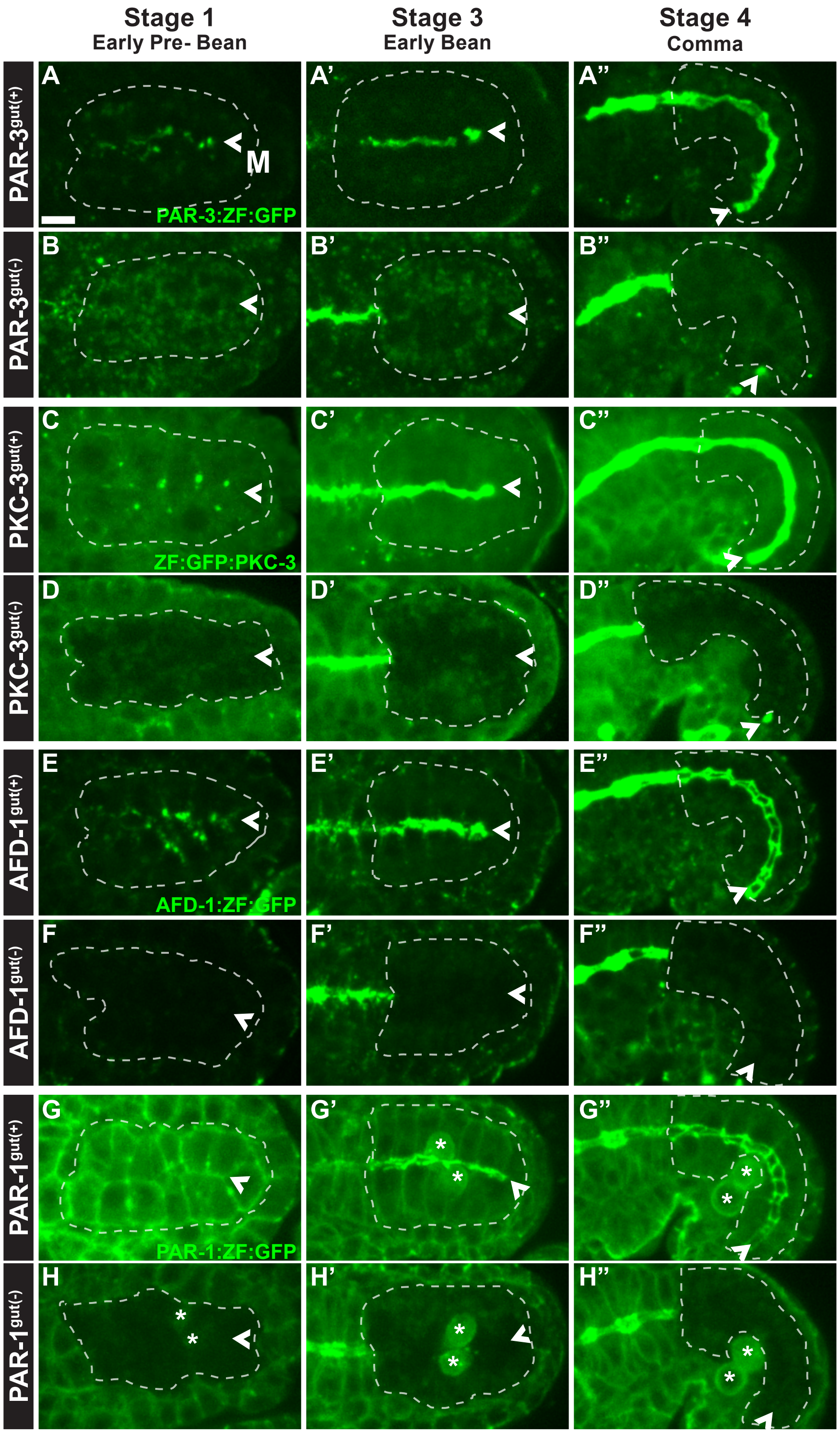
Stage 1
Early Pre- Bean
Stage 3
Early Bean
Stage 4
Comma
PAR-3gut(+)
PAR-3gut(-)
PKC-3gut(+)
PKC-3gut(-)
AFD-1gut(+)
AFD-1gut(-)
PAR-1gut(+)
PAR-1gut(-)
A
A'
A"
M
PAR-3:ZF:GFP
B
B'
B"
C
C'
C"
ZF:GFP:PKC-3
D
D'
D"
E
E'
E"
AFD-1:ZF:GFP
F
F'
F"
G
G'
G"
PAR-1:ZF:GFP
H
H'
H"

Fig. S4. Intestine specific ZIF-1 mediated protein degradation. A-H) Dorsolateral images of embryos expressing endogenously tagged proteins as indicated with (gut(-)) or without (gut(+)) intestine specific ZIF-1 expression in Stage 1, Stage 3, and Stage 4 embryos. All images are maximum intensity projections from live imaging. Intestines outlined by white dashed lines, midlines indicated by arrowheads. Enlarged versions of boxed regions shown to right. Scale bar = 5 μm for panels and 2 μm for boxed insets. Asterisks mark germ cells.

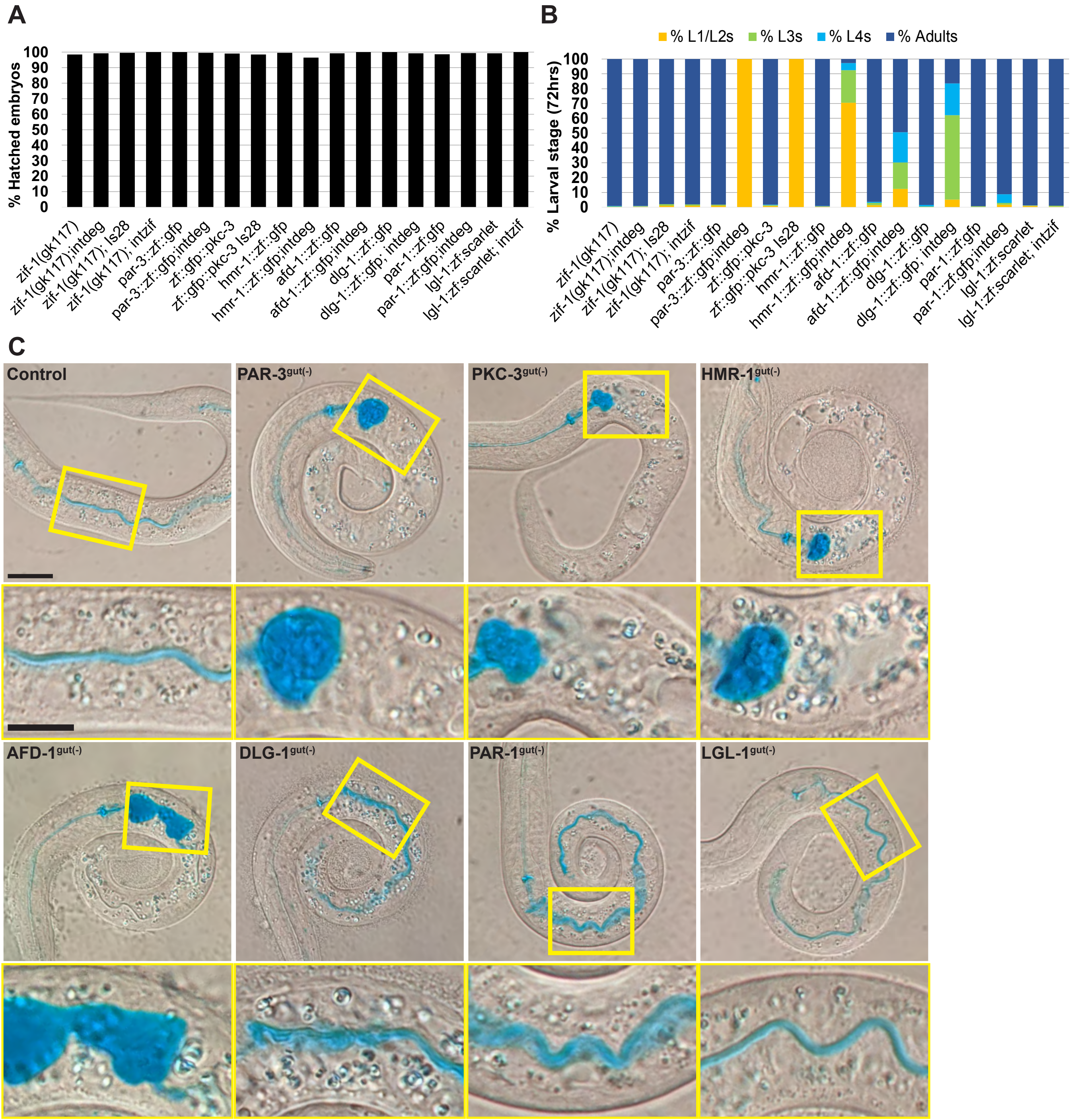

Development • Supplementary information

Fig. S5. Intestinal polarity proteins are not required for embryonic survival but are differentially required for intestinal structure and function and larval growth. A) Percentage of hatched worms 48 hours after egg lay for control (*zif-1(gk117),* n= 1267*; zif-1(gk117);intDeg,* n=698; *zif-1(gk117);Is28,* n=201; *zif-1(gk117);intZif,* n=103; *par-3::zf::gfp*, n=290; *zf::gfp::pkc-3,* n=229*; hmr-1::zf::gfp,* n=217*; afd-1::zf::gfp,* n=138; *dlg-1::zf::gfp,* n=197; *par-1::zf::gfp,* n=149; *lgl-1::zf::scarlet,* n=251*)* or intestine specific depletion of indicated protein (*par-3::zf::gfp;intDeg,* n=205*; zf::gfp::pkc-3 Is28,* n=134; *hmr-1::zf::gfp;intDeg*, n=193; *afd-1::zf::gfp;intDeg*, n=149*; dlg-1::zf::gfp;intDeg,* n=119; *par-1::zf::gfp;intDeg,* n=172; *lgl-1::zf::scarlet,* n=269*).* B) Percentage of worms at the L1/L2, L3, or L4 larval or adult stage 72 hours after egg lay for control (*zif-1(gk117),* n=1,278; *zif-1(gk117);intDeg,* n=696*; zif-1(gk117);Is28*, n=200; *zif-1(gk117);intZif,* n=102*; par-3::zf::gfp,* n=289*; zf::gfp::pkc-3,* n=229*; hmr-1::zf::gfp,* n=216*; afd-1::zf::gfp,* n=137*; dlg-1::zf::gfp,* n= 197*; par-1::zf::gfp,* n=147*; lgl-1::zf::scarlet,* n=249*)* or intestine specific depletion of indicated protein (*par-3::zf::gfp;intDeg,* n=198*; zf::gfp::pkc-3 Is28,* n=132*; hmr-1::zf::gfp;intDeg,* n=186*; afd-1::zf::gfp;intDeg,* n=146*; dlg-1::zf::gfp;intDeg*, n=116*; par-1::zf::gfp;intDeg,* n=171*; lgl-1::zf::scarlet,* n=269*)*. C) Representative DIC images of live worms fed blue food coloring showing indicated genotype, with higher magnification views of boxed regions shown below. Scale bar = 20 µm for panels and 10 µm for boxed insets.

Development • Supplementary information

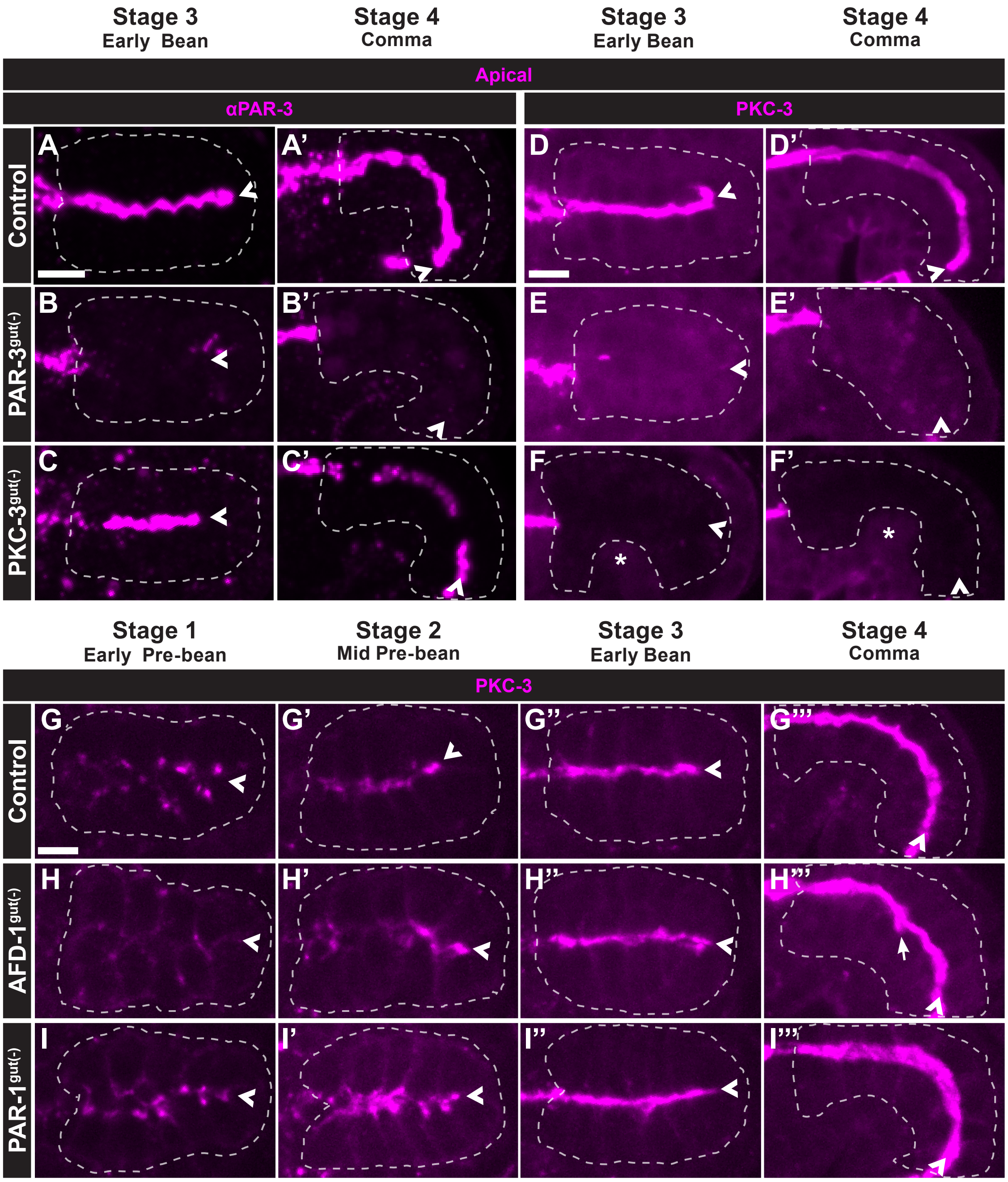

Development • Supplementary information

Fig. S6. PAR-3, PKC-3, AFD-1, and PAR-1 play different roles in apical polarity establishment and maintenance. A-C’) Dorsal (Stage 3) or dorsolateral (Stage 4) images of fixed control (A, A’, (Stage 3, n=8; Stage 4, n=19), PAR-3$^{gut(-)}$ (B, B’, (Stage 3, n=4; Stage 4, n=10), or PKC-3$^{gut(-)}$ (C,C’, (Stage 3, n=6,; Stage 4, n=5)), embryos immunostained for PAR-3. D-I’’’) Dorsal (Stage 1-3) or dorsolateral (Stage 4) live images of PKC-3 localization in control (D,D’ (Stage 3, n=;3 Stage 4, n=5), G-G’’’, (Stage 1, n=4; Stage 2, n=3; Stage 3, n=6; Stage 4, n=6)), PAR-3$^{gut(-)}$ (E,E’, (Stage 1, n=2; Stage 3, n=4)), PKC-3$^{gut(-)}$ (F,F’, (Stage 3, n=6; Stage 4, n=2)), AFD-1$^{gut(-)}$ (H-H’’’, (Stage 1, n=10; Stage 2, n=4; Stage 3, n=4; Stage 4, n=10)), or Par-1$^{gut(-)}$ (I-I’’’, (Stage 1, n=2; Stage 2, n=6; Stage 3, n=8, Stage 4, n=14)) embryos. All images are maximum intensity projections from live imaging. Intestines outlined by white dashed lines, midlines indicated by arrowheads. Arrow in H’’’ indicates slight extensions of apical surface in AFD-1$^{gut(-)}$ worms. Scale bar = 5 μm.

Development • Supplementary information

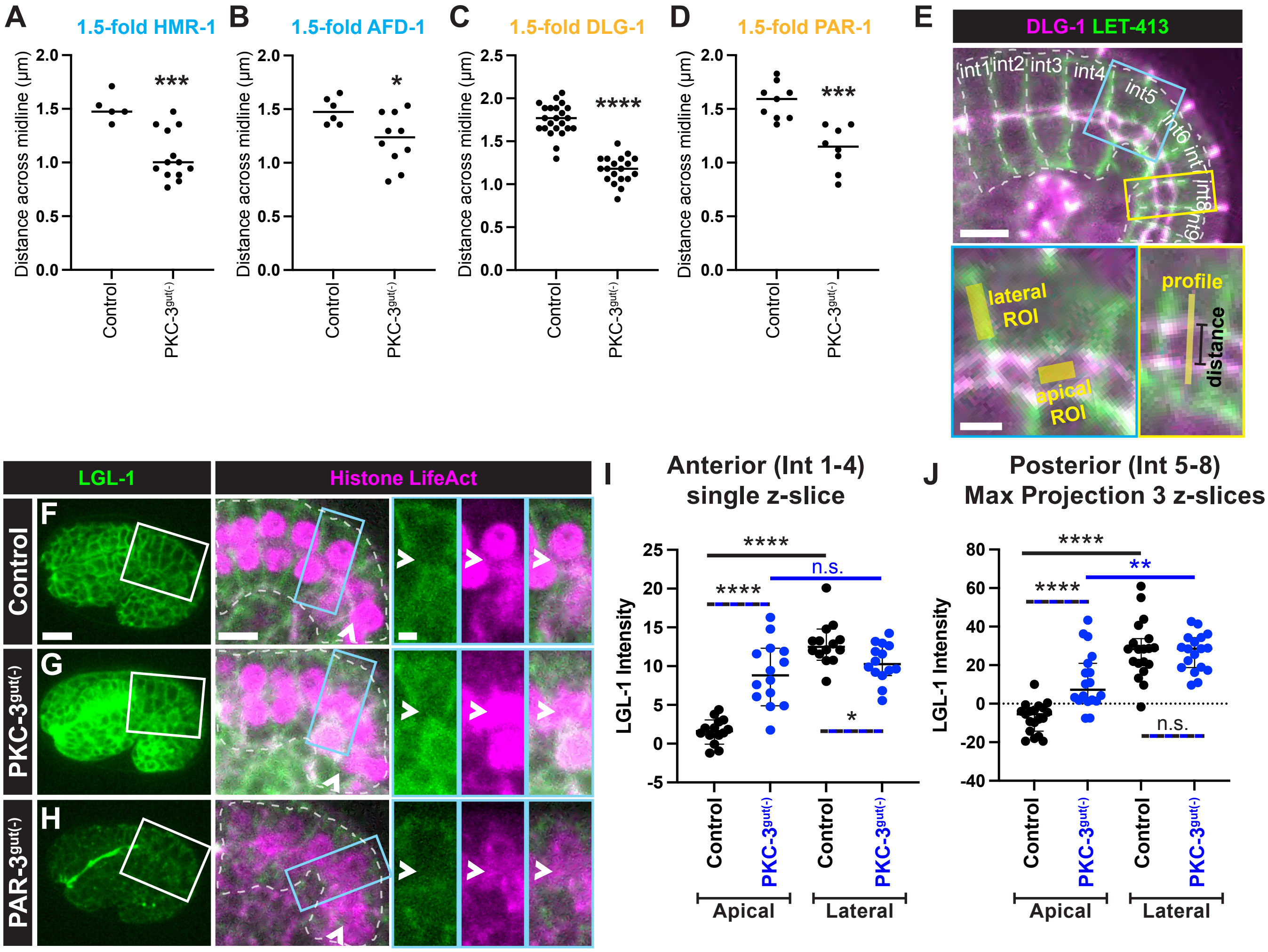

Fig. S7. PKC-3 is required for junctional separation and exclusion of basolateral proteins from the apical surface. A-D) Scatter dot plots showing the separation of the junctions across the midline in control and PKC-3gut(-) 1.5- embryos for A) HMR-1 (control n=5, PKC-3gut(-) =13), B) AFD-1 (control n=6, PKC-3gut(-) =10), C) DLG-1(control n=23, PKC-3gut(-) =19), and D) PAR-1 (control n=9, PKC-3gut(-) =8). E) Intestinal structure in 1.5-fold control embryo and sites of measurements for junction separation for A-D, and apical and lateral signal intensities in I-J. F-H) LGL-1 and F-actin localization in F) Control (n=14), G) PKC-3gut(-) (n=14), and H) PAR-3gut(-) (n=24) 1.5-fold embryos. Note in H the decreased LGL-1 localization outside of the intestine in the embryo suggesting possible synthetic interactions between tagged LGL-1 with tagged PAR-3. I-J) Scatter dot plots of the apical and lateral LGL-1 signal intensity (signal – cytoplasmic signal) for control (n=14) and PKC-3gut(-) (n=14) 1.5-fold embryos when measured I) in the anterior portion of the intestine (Int 1-4) from a single z-slice as was done for PAR-6gut(-) analysis in Sallee et al., 2021 or J) from a 3 z-slice Maximum projection from the posterior part of the intestine (Int 5-8). Statistical analysis: Student's t-test. *p<0.05, **p<0.01, ***p<0.001, ****p<0.0001. Scale bar = 10 μm for whole embryo views, 5 μm for intestine views (dashed lines surrounding intestine), and 2 μm for cellular views.

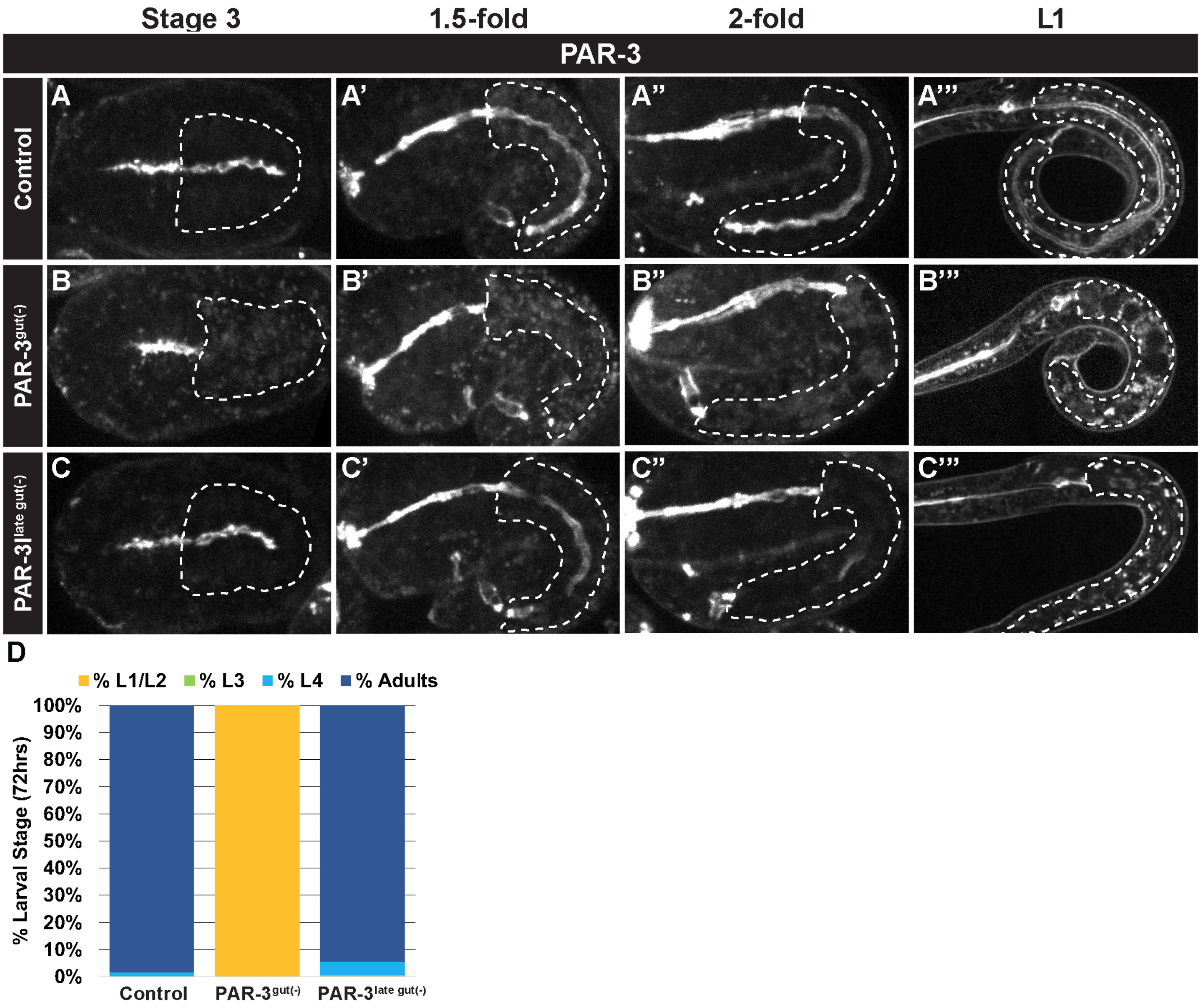

Development • Supplementary information

Fig. S8. PAR-3 is not required for polarity maintenance. A-C''') Localization of endogenously zf:gfp tagged PAR-3 in A-A''') Control, B-B''') PAR-3$^{gut(-)}$, and C-C''') PAR-3$^{late\ gut(-)}$ embryos and L1 larvae. A-A''') PAR-3 localizes continuously along the apical surface in Control embryos (Stage 3 n=9, 1.5-fold n=11, 2-fold n=8, L1 n=6). B-B''') PAR-3 is depleted from the intestine prior to polarity establishment in PAR-3$^{gut(-)}$ worms (Stage 3 n=5, 1.5-fold n=6, 2-fold n=6, L1 n=10). C-C''') In PAR-3$^{late\ gut(-)}$ worms, PAR-3 is present during polarity establishment in the intestine, begins to be degraded in 1.5- 2-fold embryos and is fully degraded in 2.5-fold and older embryos (Stage 3 n=11, 1.5-fold n=11, 2-fold n=13, L1 n=6). D) Percentage of worms at the L1/L2, L3, or L4 larval or adult stage 72 hours after egg lay for Control (*par-3:zf:gfp* n=371), PAR-3$^{gut(-)}$ (*par-3::zf::gfp;Is28(elt-2p:zif-1),* n=151), or PAR-3$^{late\ gut(-)}$ (*par-3::zf::gfp;Si4(asp-1p:zif-1)*, n=253).

Development • Supplementary information

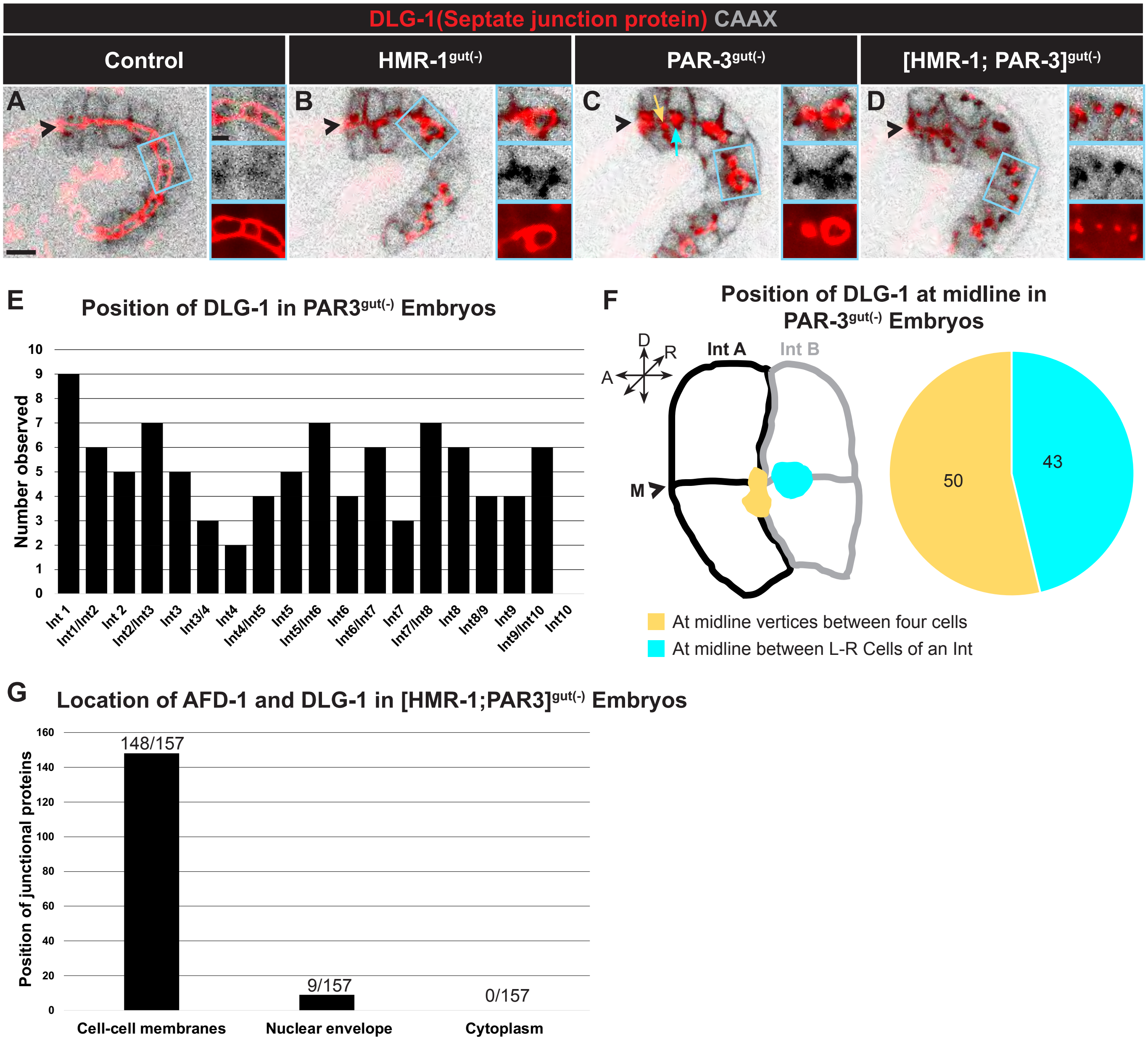

Fig. S9. Junctional proteins localize to cell-cell membranes in PAR-3$^{gut(-)}$ and [HMR-1;PAR-3]$^{gut(-)}$ embryos. A-D) DLG-1 localization relative to cell membranes in single z-slice images from A) Control (n=4), B) HMR-1$^{gut(-)}$ (n=9), C) PAR-3$^{gut(-)}$ (n=5), and D) [HMR-1;PAR-3]$^{gut(-)}$ (n=5) 1.5-fold embryos. E) Quantification of the position of DLG-1 structures relative to cell-cell contacts in PAR-3$^{gut(-)}$ Comma (n=3) and 1.5-fold (n=5) embryos. Scale bar = 5 µm for panels and 2 µm for boxed insets. F) Left: Schematic of two neighboring Anterior-Posterior (A-P) intestinal rings (Ints) with depiction of DLG-1 at the vertex between the four A-P and Left-Right (L-R) neighboring cells (gold) or only between the left and right cells of an Int (cyan). Right: Total number of DLG-1 structures observed at these positions from the embryos quantified in E. Gold and cyan arrows in C show examples of each structure. G) Location of AFD-1 and DLG- 1 puncta (157 total) relative to cell membranes in [HMR-1;PAR-3]$^{gut(-)}$ 1.5-fold embryos (n=5).

Development • Supplementary information

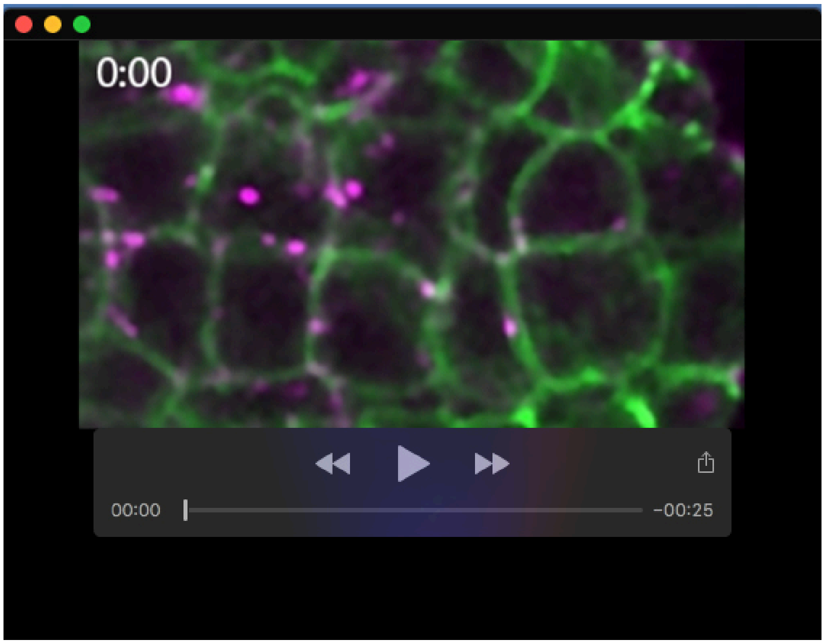

Movie 1. Dorsal view of LET-413 (green) and PAR-3 (magenta) localization from Stage 1- 2 during intestinal polarization.

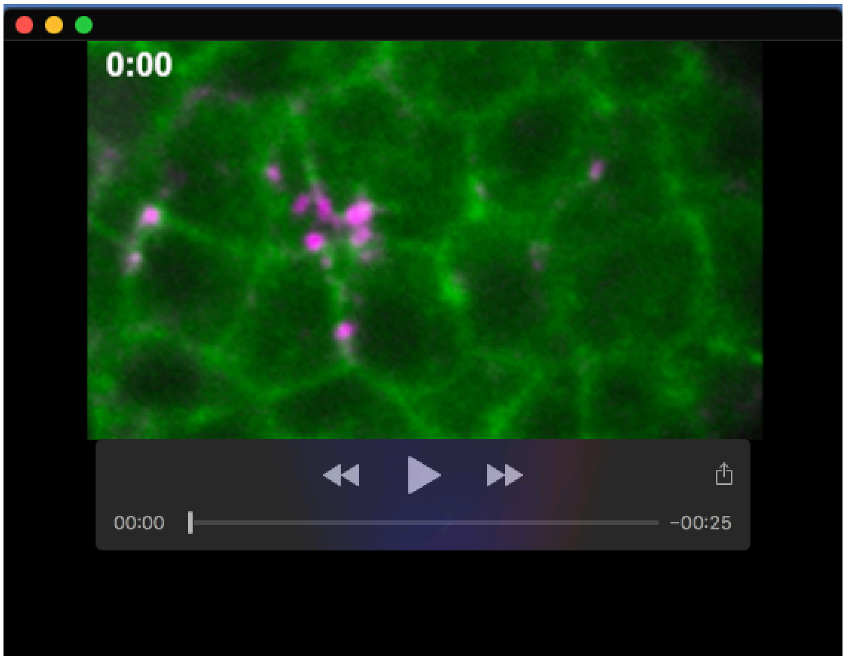

Movie 2. Dorsal view of PAR-1 (green) and PAR-3 (magenta) from Stage 1-2 during intestinal polarization.

Development • Supplementary information

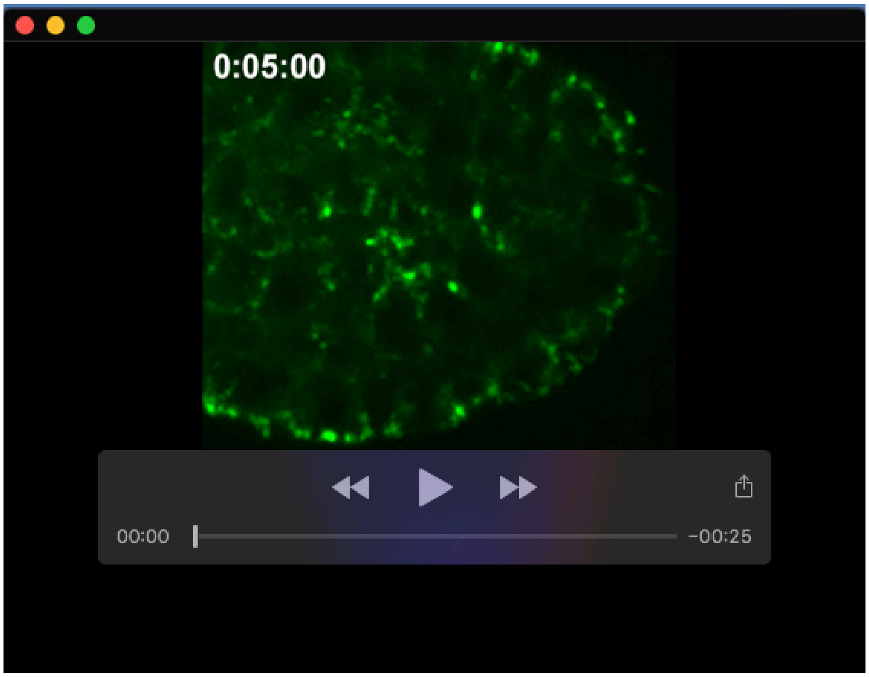

Movie 3. Dorsal view of AFD-1 (green) localization from Stage 1- 3 during intestinal polarization.

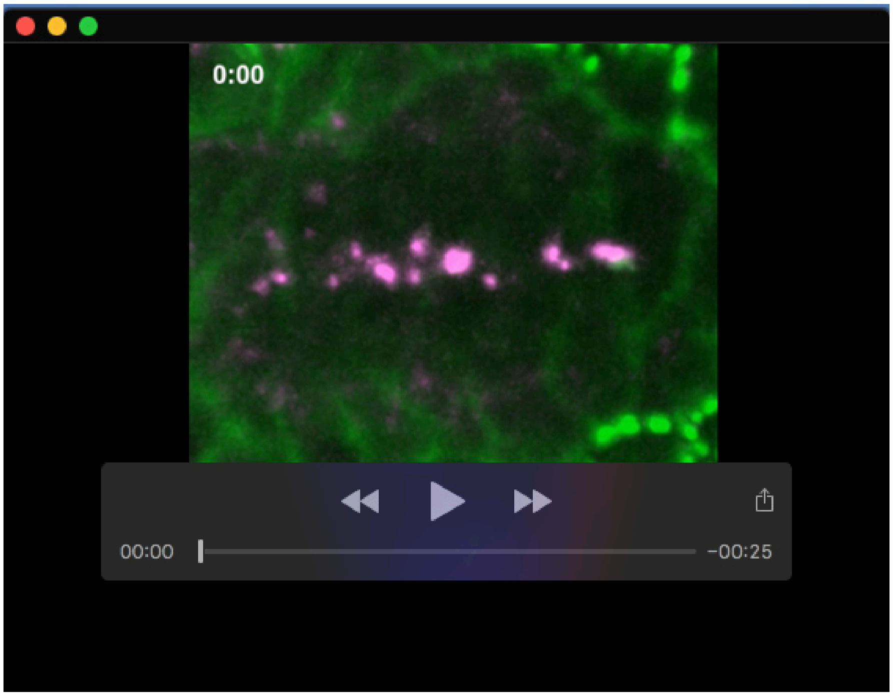

Movie 4. Dorsal view of DLG-1 (green) and PAR-3 (magenta) localization from late stage 1 through stage 2.5 during intestinal polarization.

Table S1. List of strains used in this study

Click here to download Table S1

Table S2. sgRNA and homology arm sequences, plasmids and primers used to generate new CRISPR alleles

Click here to download Table S2

Supplemental References

Beatty, A., Morton, D. and Kemphues, K. (2010). The C. elegans homolog of Drosophila Lethal giant larvae functions redundantly with PAR-2 to maintain polarity in the early embryo. *Development* **137**, 3995-4004.

Heppert, J. K., Dickinson, D. J., Pani, A. M., Higgins, C. D., Steward, A., Ahringer, J., Kuhn, J. R. and Goldstein, B. (2016). Comparative assessment of fluorescent proteins for in vivo imaging in an animal model system. *Mol Biol Cell* **27**, 3385-3394.

Huang, J., Wang, H., Chen, Y., Wang, X. and Zhang, H. (2012). Residual body removal during spermatogenesis in C. elegans requires genes that mediate cell corpse clearance. *Development* **139**, 4613-4622.

Lee, J., Magescas, J., Fetter, R. D., Feldman, J. L. and Shen, K. (2021). Inherited apicobasal polarity defines the key features of axon-dendrite polarity in a sensory neuron. *Curr Biol* **31**, 3768-3783 e3763.

Legouis, R., Gansmuller, A., Sookhareea, S., Bosher, J. M., Baillie, D. L. and Labouesse, M. (2000). LET-413 is a basolateral protein required for the assembly of adherens junctions in Caenorhabditis elegans. *Nat Cell Biol* **2**, 415-422.

MacQueen, A. J., Baggett, J. J., Perumov, N., Bauer, R. A., Januszewski, T., Schriefer, L. and Waddle, J. A. (2005). ACT-5 is an essential Caenorhabditis elegans actin required for intestinal microvilli formation. *Mol Biol Cell* **16**, 3247-3259.

Marston, D. J., Higgins, C. D., Peters, K. A., Cupp, T. D., Dickinson, D. J., Pani, A. M., Moore, R. P., Cox, A. H., Kiehart, D. P. and Goldstein, B. (2016). MRCK-1 Drives Apical Constriction in C. elegans by Linking Developmental Patterning to Force Generation. *Curr Biol* **26**, 2079-2089.

Montoyo-Rosario, J. G., Armenti, S. T., Zilberman, Y. and Nance, J. (2020). The Role of pkc-3 and Genetic Suppressors in Caenorhabditis elegans Epithelial Cell Junction Formation. *Genetics* **214**, 941-959.

Nonet, M. L. (2020). Efficient Transgenesis in Caenorhabditis elegans Using Flp Recombinase-Mediated Cassette Exchange. *Genetics* **215**, 903-921.

Rodriguez, J., Peglion, F., Martin, J., Hubatsch, L., Reich, J., Hirani, N., Gubieda, A. G., Roffey, J., Fernandes, A. R., Johnson, D. S., et al. (2017). aPKC cycles between functionally distinct PAR protein assemblies to drive cell polarity. *Developmental Cell* **42**, 400-415.

Sallee, M. D., Pickett, M. A. and Feldman, J. L. (2021). Apical PAR complex proteins protect against programmed epithelial assaults to create a continuous and functional intestinal lumen. *Elife* **10**.

Sanchez, A. D., Branon, T. C., Cote, L. E., Papagiannakis, A., Liang, X., Pickett, M. A., Shen, K., Jacobs-Wagner, C., Ting, A. Y. and Feldman, J. L. (2021). Proximity labeling reveals non-centrosomal microtubule-organizing center components required for microtubule growth and localization. *Curr Biol* **31**, 3586-3600 e3511.

Waaijers, S., Ramalho, J. J., Koorman, T., Kruse, E. and Boxem, M. (2015). The C. elegans Crumbs family contains a CRB3 homolog and is not essential for viability. *Biol Open* **4**, 276-284.
