# Supplementary material for: Separable mechanisms drive local and global polarity establishment in the Caenorhabditis elegans intestinal epithelium
Source: Development. 2022 Nov 16;149(22):dev200325. doi: 10.1242/dev.200325 (PMC9845746; doi:10.1242/dev.200325)
Supplement: Supplementary file 1 [file develop-149-200325_review_history.pdf]

Separable mechanisms drive local and global polarity establishment in the *Caenorhabditis elegans* intestinal epithelium

Melissa A. Pickett, Maria D. Sallee, Lauren Cote, Victor F. Naturale, Deniz Akpinaroglu, Joo Lee, Kang Shen and Jessica L. Feldman

DOI: 10.1242/dev.200325

Editor: Thomas Lecuit

Review timeline

Original submission:	2 November 2021
Editorial decision:	13 January 2022
First revision received:	15 August 2022
Accepted:	6 October 2022

Original submission

First decision letter

MS ID#: DEVELOP/2021/200325

MS TITLE: Separable mechanisms drive local and global polarity establishment in the C. elegans intestinal epithelium

AUTHORS: Melissa A Pickett, Maria D. Sallee, Victor F. Naturale, Deniz Akpinaroglu, Joo Lee, Kang Shen, and Jessica Feldman

I sincerely apologize for the long delay before coming back to you. I have now received all the referees reports on the above manuscript, and have reached a decision. The referees' comments are appended below, or you can access them online: please go to BenchPress and click on the 'Manuscripts with Decisions' queue in the Author Area.

The overall evaluation is positive and we would like to publish a revised manuscript in Development, provided that the referees' comments can be satisfactorily addressed. Please attend to all of the reviewers' comments in your revised manuscript and detail them in your point-by-point response. If you do not agree with any of their criticisms or suggestions explain clearly why this is so.

We are aware that you may currently be unable to access the lab to undertake experimental revisions. If it would be helpful, we encourage you to contact us to discuss your revision in greater detail. Please send us a point-by-point response indicating where you are able to address concerns raised (either experimentally or by changes to the text) and where you will not be able to do so within the normal timeframe of a revision. We will then provide further guidance. Please also note that we are happy to extend revision timeframes as necessary.

Reviewer 1

Advance summary and potential significance to field

Melissa Picket and collaborators have used C. elegans to examine the fine distribution of eight key polarity and junctional proteins during development, as well as their requirements to establish a

© 2022. Published by The Company of Biologists under the terms of the Creative Commons Attribution License (https://creativecommons.org/licenses/by/4.0/).

polarized intestinal epithelium using gut-specific depletion of several markers. Their precise mapping shows that proteins that will eventually become basolateral (LET-413, LGL-1, PAR-1, DLG-1) can be found at early stages of polarization at the midline with apical markers (PAR-3, PKC-3, HMR-1, AFD-1). They found that these markers are in order of requirements for survival and polarization PAR-3 = PKC-3 ≈ HMR-1 > DLG-1 ≈ AFD-1 >> PAR-1 ≈ LGL-1. One notable missing factor in this list is LET-413, which was not tested using the same strategy. They further found that PAR-3 requirement is more important than that of PKC-3 for gut polarization, since the adherens junction proteins HMR-1 and AFD-1 could assemble in the absence of PKC-3 but not of PAR-3. Finally, they report that PAR-3 is not required for initial cellular polarization, but is essential for global polarization across the tissue, and that the combined absence of PAR-3 with E-cadherin/HMR-1 prevents local cellular polarization.

The finding that PAR-3 has an essential role for global polarization, but not for local polarization, at least alone, is an interesting conceptual advance. Their ability to spatially target a gut-specific depletion of seven markers really represents a great tool, and the subsequent detailed study of the different marker requirements is nice.

Comments for the author

Overall the conclusions are solid and the work is well done, although some improvements could be brought (see below), hence I strongly favor publication in Development once comments have been addressed.

Major comments

1. In several cases, I was not entirely convinced by the statements. For instance when they say (line 164-165) that LGL-1 and LET-413 colocalized with PAR-3, or when they examine the distance between various markers at the lumen in Figure 2. I am concerned with their resolution using regular spinning disk confocal microscopy, and I think that super-resolution microscopy would be better to confirm their conclusions.
2. It would be ideal to report what happens in LET-413(gut-) embryos, since LET-413 and its fly homologue Scribble play an important role in setting up epithelial polarity. Will it be as severe as in PAR-3(gut-)/PKC-3(gut-) or more like in DLG-1(gut-) embryos? Was there a technical difficulty in generating those embryos?

Minor

3. Please indicate how embryo staging was done, which marker was used to decide whether a particular embryo was of stage 1/2/3/4.
4. Lines 218-220: I think that it has previously been clearly proposed that the zonula adherens in C. elegans splits into an AJ and an SJ (though the term SJ has not been used so far), so the statement in those lines should be toned down and reference to the appropriate papers should be made (Hardin, Labouesse, Rongo labs).
5. Lines 290-291: please point out in Fig S5 what you mean by “Minor extensions”.
6. Line 298: the apical surface expansion mentioned here should ideally be quantified.
7. Fig 5B’/B’’: the exclusion of LET-413 from the lumen is not that clear. How frequently has it been observed? Has a luminal/apical marker also been used to ascertain lumen exclusion?
8. Line 424: could you please clarify what you mean when using the words “align their polarity axis”. Do you have in mind planar polarity?

<u>Reviewer 2</u>

Advance summary and potential significance to field

Examining polarization of C. elegans intestinal cells, Pickett and colleagues provide evidence for genetically and temporally separable stages of global and local cell polarization. Using an impressive suite of endogenously tagged polarity proteins and conditional versions that can be degraded using the ZF degron system, they query the cellular function of numerous apical, junctional, and basolateral proteins. These experiments define a role for PAR-3 in global polarization, although at later stages the authors provide evidence that clusters of cells can form

© 2022. Published by The Company of Biologists under the terms of the Creative Commons Attribution License (https://creativecommons.org/licenses/by/4.0/).

organized apical-basal domains that are not coordinated across the tissue. Double depletion of PAR-3 and the cadherin HMR-1 perturbs the formation of these clusters suggesting that AJ proteins play key roles in their formation.

Epithelial cell polarization in C. elegans has been examined in numerous prior studies, most of which relied on previous-generation reagents or methods (antibodies, transgenes, global knockdown). The major strength of this manuscript is in the nearly comprehensive set of proteins that are examined at the level of localization and function using endogenously tagged genes. Many of the findings support previously published ones although there are numerous new contributions. Most significant are the experiments detailing the phenotype that results from PAR-3 depletion, and the finding that HMR-1 plays an instrumental role in the local polarity that develops in PAR-3(-) intestines.

Comments for the author

My one major experimental suggestion, given that the authors have already created the tools to address it and it would be a significant contribution, would be to test their hypothesis (raised in the discussion) that PAR-3 is only needed when polarity is being established. This could likely be accomplished easily by using a heatshock promoter to express ZIF-1 at a few different stages of polarization. Although a separate study in larvae suggests that PAR-3 is not needed after embryogenesis, this was determined using a different method (auxin-inducible degradation) and no effort was made to establish when PAR-3 function was required.
Otherwise, I have only minor suggestions. The paper is written and presented well, and I feel it would be well received by readers of Development.
Minor comments –
Abstract line 50
“Despite belonging to the same apical protein complex” – this is an oversimplification; PAR-3, PAR-6 and PKC-3 do not always form a tripartite complex, and as the authors and others have shown, PKC-3 and PAR-3 sort out from one another after polarity establishment
Results line 151 It would help those who are not familiar with C. elegans embryos to indicate in the Figure that the embryo in A’’’ has rotated 90 degrees to its side. The cells in the images are in a very different orientation at this stage.
Line 155
“spread within each cell” - do the authors know from live imaging that the foci actually spread? Or are more nucleated, or do clusters fragment? I would change the wording here unless spreading can be confirmed.
Line 167 par-1 is a very complicated gene with many isoforms predicted. It would be helpful to show for par-1, and other genes tagged, where the tags are placed and whether there are predicted isoforms that might not include them (perhaps in a Supplemental Figure illustration); without this information it is hard to interpret phenotypes and localization patterns. If there is evidence that some isoforms might not include the tags, this should be stated in the results and interpretations qualified. Although this information could be gleaned by the eager from the methods, that is asking a lot of the casual reader!
Line 196 The authors should more explicitly point out when their results confirm previous observations and when they are new. This applies to the more basal localization of DLG-1 compared to AJ proteins in this section, as well as numerous findings throughout the paper. It is hard to glean this information from a cited reference if the authors do not explicitly tell the readers that their finding agrees with the prior finding.
Line 215 The more basal localization of PAR-1 compared to DLG-1 would be a new finding. However, from the data presented, I was not convinced – statistical significance was not as strong as the other proteins that are known to have separate localization domains, and in contrast to these pairs, there were several examples of embryos where PAR-1 localized more apical than DLG-1 (although on average not). The authors should make efforts to show that this finding is robust and reproducible, using similar imaging methods or super-resolution methods.
Line 245 What might explain the discrepancy between 30% of hmr-1(-) larvae growing beyond L1 and the 88% that have an occluded lumen? From the description, I would imagine an occluded lumen to prevent growth.
Line 311
“but these junctions appeared to be closer together in Stage 4 PKC-3…” this observation should be quantified from the existing data.

© 2022. Published by The Company of Biologists under the terms of the Creative Commons Attribution License (https://creativecommons.org/licenses/by/4.0/).

Line 360 The finding that LET-413 does not require PKC-3 to be excluded from the apical domain is surprising and new. Since in the early embryo PKC-3 phosphorylation of LGL-1 is thought to be required for LGL-1 localization, it would be helpful to know if LGL-1 localization is also independent of PKC-3 in the intestine.
Line 394
"…junctional rings were completely abolished…" this phenotype should be quantified.
Line 407
"polarity may still arise in the absence of both PAR-3 and HMR-1" The caveat that double depletion may not be complete, and that the phenotype is hypomorphic, should be raised here.
Figure 1 1A: The figure legend did not include what was depicted in magenta. Although I'm assuming that this is PAR-3 from the rest of this figure, this is not obvious.
1F-1I: Perhaps drawing a yellow transparent line (like that shown in Figure 2) across the midline will help readers understand where these measurements are being taken.
Figure 2:
2D''-2H'': The authors include a graph that measures fluorescence intensity across the midline in Supp Fig 2D that seems easier to understand the point that proteins belonging to separate polarity domains localize differently. Perhaps the authors should consider presenting this data in a similar fashion.
Figure 2/4: The authors show in Figure 2 that PAR-1 localizes more basally than DLG-1 (but see comments above) although in Figure 4 the authors then refer to PAR-1 as a septate like junction protein.
Figure 3 3A vs 3B: While the "% Adult" phenotype quantification comes from the top of the bar graph in 3A, the "%
Normal" phenotype in 3B comes from the bottom of the bar graph, making the comparison between organismal viability and intestinal function harder to compare.

Reviewer 3

Advance summary and potential significance to field

This paper characterizes how the cell polarity network operates on the cell & tissue levels during gut formation in C. elegans by using tissue specific protein degradation techniques and live imaging. The authors show that apical identity is specified first in the gut, upstream of basolateral segregation, and show that PAR-3 knockdown is particularly disruptive to embryonic gut development. Interestingly, the authors find that loss of PAR-3 causes a discontinuous gut with pockets of correctly polarized tissue that form cysts with apparently normal apical and junctional protein localization. When both polarity (PAR-3) and junctions (HMR-1) are perturbed, these polarized edemas go away, indicating that even when tissue polarity is lost as is the case in PAR-3 (-), HMR-1 is still part of a pathway that allows for correct polarity in individual cells.

This manuscript is particularly strong in careful staging and imaging of gut development, which provides a clear and thorough overview of the developmental process and its genetic requirements. Also, the connection between embryonic gut morphology, larval arrest, and larval gut morphology provide a solid understanding of how different polarity/junctional proteins contribute to gut development. The detailed analysis of the PAR-3(-) phenotype and subsequent PAR-3/HMR-1 mutants are particularly interesting. Overall, the manuscript is well presented and we have no major concerns. Below are minor suggestions for improvement, including adding some data that the authors likely already have obtained -- no new experiments are necessary.

Comments for the author

Minor comments:

- How PAR-3 and HMR-1 carry out their redundant/parallel functions mechanistically is not within the scope of this paper, but a few additional images/analyses might help to clarify the exact nature of the phenotype in HMR-1(-)/PAR-3(-) intestines.
- We were curious whether knockdown of HMR-1 in the PAR-3(-) background eliminates polarity via loss of a specific mechanism at junctions, or if instead there is polarity lost

© 2022. Published by The Company of Biologists under the terms of the Creative Commons Attribution License (https://creativecommons.org/licenses/by/4.0/).

because there are no junctions/cell contacts for the polarization to occur at. Can the authors provide any insight into this question?

- In Figure 7F-F'', it was unclear where the AFD-1/DLG-1 puncta are localized within the cells - are they on membranes, cytoplasmic, random? Including brightfield images and pointing out cell membranes would help orient these puncta within the tissue.
- We found the detailed staging and timeline of polarization laid out in figure 1 to be thorough and helpful to understanding the process of gut polarization. However, figure 7 only contains images of later stages. Adding images of early stages in the double mutant (hmr-1; par-3) would be useful in connecting the phenotypes across figures.
- We were curious if the gut lumen edemas, found in the various mutants, formed in stereotyped locations between pairs of intestinal cells or stochastically. For example, do they form exclusively at midlines where the lumen swells, or do some occur as ectopic lumens forming between lateral cell-cell contacts? Scoring positions of edemas could help generate hypotheses about which junctions are maintained and what exactly goes wrong when edemas form.
- There is some minor redundancy in the data shown here and in the authors' earlier work (Sallee et al. 2021):
- The aPKC gut (-) phenotypes shown in Figure 3 of this manuscript were reported in Figure 6 of Sallee et al., although they are compared to a wider variety of genetic backgrounds here. This is not really an issue, but it would be helpful if the authors would more specifically refer back to their previous work in order to put the current results in context.
- Mislocalization of LET-413 and DLG-1, reported for aPKC gut(-) and PAR-3 gut (-) in Figure 5 of this manuscript, was shown previously for PAR-6 gut(-) in Figure 5 of Sallee et al. Strictly speaking, these data are not overlapping, since different proteins were depleted, but it would be shocking if the PAR-6 and aPKC phenotypes were different since these two proteins form an obligate heterodimer. Again, it would be helpful for the reader if the results from Sallee et al. were specifically mentioned and discussed in the context of Figure 5.
- Figure 1: A supplemental movie would help with visualizing puncta movement during polarity establishment, if feasible.
- Figure 2 D-H'': It's unclear whether the resolution of the images allows for the distance measurements performed. From the images, the proteins look colocalized, and comparison of proteins where distance was significant (such as D) look similar to proteins where distance was statistically insignificant (such as E). Because the precise localization of these junctional proteins isn't critical to the findings of the rest of the paper, we suggest omitting the distance quantification.
- We would prefer to see the PKC-3 and PAR-3 data from figure S5 included in the main Figure 5 - these data are interesting and important because they help to clearly establish the hierarchy of polarity proteins in this tissue.
- Discussion: The authors write "The kinase activity of PKC-3 is thought to be inactive when in complex with PAR-3, and one role of PAR-3 may be to keep PKC-3 in an inactive state (Soriano et al., 2016)." However, the conclusions of Soriano et al. have been challenged (Holly & Prehoda, 2019). We would urge the authors to be cautious about taking sides in this debate in the absence of direct evidence from C. elegans.

First revision

Author response to reviewers' comments

Please find enclosed our revised manuscript, "Separable mechanisms drive local and global polarity establishment in the C. elegans intestinal epithelium." We appreciate your patience in our resubmission that was delayed due to numerous medical complications.

We were happy to see that the reviewers were generally enthusiastic about our work and asked for mostly minor revisions. In response to reviewer comments, we have added new data and quantification in 2 new main figure panels, 4 new supplemental figures (Figure S3, S7. S8. S9), and 4 new supplemental videos:

© 2022. Published by The Company of Biologists under the terms of the Creative Commons Attribution License (https://creativecommons.org/licenses/by/4.0/).

Reviewer 1 Comments for the author

Overall the conclusions are solid and the work is well done, although some improvements could be brought (see below), hence I strongly favor publication in Development once comments have been addressed.

Thank you!

Major comments

[1.In]1.In several cases, I was not entirely convinced by the statements. For instance when they say (line 164-165) that LGL-1 and LET-413 colocalized with PAR-3, or when they examine the distance between various markers at the lumen in Figure 2. I am concerned with their resolution using regular spinning disk confocal microscopy, and I think that super-resolution microscopy would be better to confirm their conclusions.

Unfortunately, super-resolution microscopy is not feasible with our existing worm strains and available super-resolution technology. Out of focus light from the very bright endogenous signal in the skin makes the reconstruction for structured illumination microscopy impossible in our hands. We acknowledge the resolution limits to co-localization analysis using confocal microscopy and have added text acknowledging these limitations on lines 162-163. Despite not using super-resolution imaging, we are still able to detect differences between junctional markers and thus higher resolution microscopy is unlikely to meaningfully alter our conclusions concerning the junctions.

[2.It]2.It would be ideal to report what happens in LET-413(gut-) embryos, since LET-413 and its fly homologue Scribble play an important role in setting up epithelial polarity. Will it be as severe as in PAR-3(gut-)/PKC-3(gut-) or more like in DLG-1(gut-) embryos? Was there a technical difficulty in generating those embryos?

We appreciate that a zf tagged version of LET-413 would be useful for degrading LET-413 specifically in the intestine for comparison with the other tagged polarity proteins in Figure 3. We were able to generate a strain in which LET-413 is endogenously tagged with zf:gfp using CRISPR/Cas9. This N-terminal tag is located at the same site as the gfp:aid:let-413 lines described in Riga et al., 2021 and Lee et al., 2021, however our strain produced high numbers of dead embryos (~40% embryonic lethality) despite backcrossing. Due to the high embryonic lethality, we were uncomfortable moving forward with this strain. We reinjected the SEC and sgRNA plasmids in an attempt to generate additional healthy zf:gfp:let-413 lines, but were unable to create any additional worm strains.

Minor

3.Please indicate how embryo staging was done, which marker was used to decide whether a particular embryo was of stage 1/2/3/4.

We have added a section to the Methods entitled “Embryo staging” Lines 656-668 to explain how embryo staging was done in more depth.

4.Lines 218-220: I think that it has previously been clearly proposed that the zonula adherens in C. elegans splits into an AJ and an SJ (though the term SJ has not been used so far), so the statement in those lines should be toned down and reference to the appropriate papers should be made (Hardin, Labouesse, Rongo labs).

We have altered the text to acknowledge the prior work from antibody staining that suggests separation within the intestinal apical junction while also emphasizing the addition our work makes to the understanding of these separate regions within the junction using endogenously tagged proteins, live imaging, and quantification Lines 209-210 and 216-218.

5.Lines 290-291: please point out in Fig S5 what you mean by “Minor extensions”.

© 2022. Published by The Company of Biologists under the terms of the Creative Commons Attribution License (https://creativecommons.org/licenses/by/4.0/).

We added an arrow pointing towards an example of an extension of the apical surface to Fig S6 H''' (previously Fig S5) and lines to describe the arrow in supplemental figure legend 6.

6.Line 298: the apical surface expansion mentioned here should ideally be quantified.

The data were quantified and were not significantly different from controls. We have edited the text to reflect this quantification on lines 290-293.

[7.Fig]7.Fig 5B'/B'': the exclusion of LET-413 from the lumen is not that clear. How frequently has it been observed? Has a luminal/apical marker also been used to ascertain lumen exclusion?

We have quantified LET-413 at the apical and lateral surfaces and now include these data in the text lines 349-351 and in figure 5 D. We used DLG-1 in 1.5-fold stage embryos to mark the edges of the apical surface and found that while LET-413 primarily localized to the lateral membranes in PKC-3gut(-) embryos, it was not excluded from the apical surface to the same degree as in controls. We describe our quantification in the Methods lines 682-709. We also found similar results investigating LGL-1, which is now included as Supplemental Figure 7.

8.Line 424: could you please clarify what you mean when using the words “align their polarity axis”. Do you have in mind planar polarity?

We mean that cells align their apico-basolateral axes relative to a common feature, in this case the intestinal midline. We have altered the text to add clarity Line 436-438.

Reviewer 2 Comments for the author

My one major experimental suggestion, given that the authors have already created the tools to address it and it would be a significant contribution, would be to test their hypothesis (raised in the discussion) that PAR-3 is only needed when polarity is being established. This could likely be accomplished easily by using a heatshock promoter to express ZIF-1 at a few different stages of polarization. Although a separate study in larvae suggests that PAR-3 is not needed after embryogenesis, this was determined using a different method (auxin-inducible degradation) and no effort was made to establish when PAR-3 function was required. Otherwise, I have only minor suggestions. The paper is written and presented well, and I feel it would be well received by readers of Development.

We thank the reviewer for this great suggestion. To address this important point, we generated a new worm strain using a promoter (asp-1p) to drive ZIF-1 expression beginning at the 1.8-fold embryonic stage and continuing through larval stages. Thus, this asp1p:ZIF-1 transgene (''late gut (-)') will degrade ZF-tagged proteins after apico-basolateral polarity establishment (Sallee et al 2021). In contrast to the larval arrest resulting from degradation of PAR-3 prior to polarity establishment, we found that degradation of PAR-3 after the 1.8-fold stage had no impact on larval growth or development, with 100% of PAR-3late gut(-) worms reaching adulthood by three days post egg lay. This result suggests that PAR-3 is only required within the intestine during polarity establishment, consistent with previously published results (Castiglioni et al, 2020). These new results have been incorporated into the paper in Supplemental Figure 8 and lines 390-396.

Minor comments –

Abstract line 50
“Despite belonging to the same apical protein complex” – this is an oversimplification; PAR-3, PAR-6 and PKC-3 do not always form a tripartite complex, and as the authors and others have shown, PKC-3 and PAR-3 sort out from one another after polarity establishment

We agree that our statement is a bit of a simplification. We have attempted to make this language more precise, line 51. We would note, however, that there is continued co-localization of PAR-3 and PKC-3 at the apical surface of the intestine following polarity establishment (Sallee et al 2021) unlike the complete separation of PAR-3 to junctions that is observed in other systems.

© 2022. Published by The Company of Biologists under the terms of the Creative Commons Attribution License (https://creativecommons.org/licenses/by/4.0/).

Results line 151
It would help those who are not familiar with C. elegans embryos to indicate in the Figure that the embryo in A''' has rotated 90 degrees to its side. The cells in the images are in a very different orientation at this stage.

We thank the reviewer for this point and have added axes to help orient our readers.

Line 155
"spread within each cell" - do the authors know from live imaging that the foci actually spread? Or are more nucleated, or do clusters fragment? I would change the wording here unless spreading can be confirmed.

This point is well taken. Although we have never done photomarking experiments to formally prove that the same protein that begins on the lateral membranes creates the continuous line of protein seen at the apical surface, numerous time lapse imaging experiments done with 2-3 minute time steps is highly suggestive of spreading (Feldman and Priess, 2012; Sallee et al., 2021). However, we also see a clear addition of new material to the surface as the final amount of endogenous fluorescent protein at the apical surface is far greater than what we see in the lateral puncta. We have changed this to read "appear to spread", now line 152. We have also included additional timelapse videos (Supplemental movies 1,2, and 4) that show the movement of PAR-3 during polarity establishment.

Line 167
par-1 is a very complicated gene with many isoforms predicted. It would be helpful to show for par-1, and other genes tagged, where the tags are placed and whether there are predicted isoforms that might not include them (perhaps in a Supplemental Figure illustration); without this information it is hard to interpret phenotypes and localization patterns. If there is evidence that some isoforms might not include the tags, this should be stated in the results and interpretations qualified. Although this information could be gleaned by the eager from the methods, that is asking a lot of the casual reader!

We agree. All predicted/known isoforms of the polarity proteins were tagged with zf:gfp as is now depicted in Supplemental Figure 3.

Line 196
The authors should more explicitly point out when their results confirm previous observations and when they are new. This applies to the more basal localization of DLG-1 compared to AJ proteins in this section, as well as numerous findings throughout the paper. It is hard to glean this information from a cited reference if the authors do not explicitly tell the readers that their finding agrees with the prior finding.

We have rewritten areas where there may have been ambiguity as to whether our results were completely new or confirm existing observations. For example, please see lines 209-210 and 216-218.

Line 215
The more basal localization of PAR-1 compared to DLG-1 would be a new finding. However, from the data presented, I was not convinced – statistical significance was not as strong as the other proteins that are known to have separate localization domains, and in contrast to these pairs, there were several examples of embryos where PAR-1 localized more apical than DLG-1 (although on average not). The authors should make efforts to show that this finding is robust and reproducible, using similar imaging methods or super-resolution methods.

As indicated above, we are unable to use our current endogenously tagged strains for super-resolution microscopy due to out of focus light in the skin that complicates the reconstruction used in structured illumination microscopy. We added to our analysis by examining the localization of the junctional portion of LET-413 to DLG-1 in the Control embryos shown in Figure 5 and found the same basal shift of LET-413 relative to DLG-1 that we observed for PAR-1. These new data are now reported in Figure 5 E and in lines 214-216 and 351-353. We hope that future work will further

© 2022. Published by The Company of Biologists under the terms of the Creative Commons Attribution License (https://creativecommons.org/licenses/by/4.0/).

elucidate the structure within the junctions, but feel that this result strengthens our conclusion that there is a more basal region of the junction.

Line 245
What might explain the discrepancy between 30% of hmr-1(-) larvae growing beyond L1 and the 88% that have an occluded lumen? From the description, I would imagine an occluded lumen to prevent growth.

We were also surprised by this observation which suggests there is something different about the cystic lumens in HMR-1gut(-) and PAR-3gut(-) or PKC-3gut(-) larvae that allows HMR-1gut(-) worms to recover. We do not understand how these animals recover and are following up on this recovery in future work.

Line 311
"but these junctions appeared to be closer together in Stage 4 PKC-3…" this observation should be quantified from the existing data.

We thank the reviewer for this suggestion. This information is now presented in Figure 5 E, and Supplemental Figure 7 A-D and is described in lines 305-307, 326-328, and 353-354.

Line 360
The finding that LET-413 does not require PKC-3 to be excluded from the apical domain is surprising and new. Since in the early embryo PKC-3 phosphorylation of LGL-1 is thought to be required for LGL-1 localization, it would be helpful to know if LGL-1 localization is also independent of PKC-3 in the intestine.

We have quantified LET-413 at the apical and lateral surfaces and now include this data in the text and Figure 5 D. We used DLG-1 in 1.5-fold stage embryos to mark the edges of the apical surface and found that while LET-413 primarily localized to the lateral membranes in PKC-3gut(-) embryos, it is not excluded from the apical surface to the same degree as in controls as described in lines 349-351 and our Methods Lines 682-709. We also found similar results investigating LGL-1, which is now included as Supplemental Figure 7 and in the text lines 355-356.

Line 394
"…junctional rings were completely abolished…" this phenotype should be quantified.

We have included the total number of embryos we have examined in figure legend 7 and the localization of DLG-1/AFD-1 puncta is quantified now in Supplemental Figure 9.

Line 407
"polarity may still arise in the absence of both PAR-3 and HMR-1" The caveat that double depletion may not be complete, and that the phenotype is hypomorphic, should be raised here.

We have added language to acknowledge this caveat on lines 418-420.

Figure 1
1A: The figure legend did not include what was depicted in magenta. Although I'm assuming that this is PAR-3 from the rest of this figure, this is not obvious.

We have added text to indicate that the magenta in part A depicts PAR-3 localization.

1F-1I: Perhaps drawing a yellow transparent line (like that shown in Figure 2) across the midline will help readers understand where these measurements are being taken.

We have added a line as suggested.

Figure 2:
2D''-2H'': The authors include a graph that measures fluorescence intensity across the midline in Supp Fig 2D that seems easier to understand the point that proteins belonging to separate polarity

© 2022. Published by The Company of Biologists under the terms of the Creative Commons Attribution License (https://creativecommons.org/licenses/by/4.0/).

domains localize differently. Perhaps the authors should consider presenting this data in a similar fashion.

We appreciate the feedback, but feel that the paired differences between junctional markers that is shown allows us to more easily represent all of the data that was analyzed than would be accomplished by showing a single representative line plot.

Figure 2/4: The authors show in Figure 2 that PAR-1 localizes more basally than DLG-1 (but see comments above) although in Figure 4 the authors then refer to PAR-1 as a septate like junction protein.

We appreciate this point. We find that both PAR-1 and LET-413 localize basal to DLG-1 suggesting that there is a more basal region of the junctions, but since LET-413 is described as contributing to septate junctions in other systems, still use the term “septate-like” to describe these more basal junctional proteins as well.

Figure 3
3A vs 3B: While the “% Adult” phenotype quantification comes from the top of the bar graph in 3A, the “% Normal” phenotype in 3B comes from the bottom of the bar graph, making the comparison between organismal viability and intestinal function harder to compare.

We appreciate this point. We have altered the order of 3B so that the %Normal phenotype is shown on the top of the bar graph to be shown in the same manner as 3A.

Reviewer 3 Comments for the author
Minor comments:

- How PAR-3 and HMR-1 carry out their redundant/parallel functions mechanistically is not within the scope of this paper, but a few additional images/analyses might help to clarify the exact nature of the phenotype in HMR-1(-)/PAR-3(-) intestines.

- We were curious whether knockdown of HMR-1 in the PAR-3(-) background eliminates polarity via loss of a specific mechanism at junctions, or if instead there is polarity lost because there are no junctions/cell contacts for the polarization to occur at. Can the authors provide any insight into this question?

Membranes and cell contacts are still present between the cells of the intestine, where polarization could theoretically occur. We additionally analyzed each z-slice from our confocal stacks to map the locations of the AFD-1/DLG-1 puncta relative to the cell membranes. This analysis is now shown and quantified in Supplemental Figure 9.

- In Figure 7F-F’’, it was unclear where the AFD-1/DLG-1 puncta are localized within the cells - are they on membranes, cytoplasmic, random? Including brightfield images and pointing out cell membranes would help orient these puncta within the tissue.

- We analyzed the locations of the AFD-1/DLG-1 puncta and find that they localize almost exclusively to cell membranes. We have also included single z-slices instead of max. projections to highlight the location of these puncta within the tissue in Supplemental Figure 9 and mentioned in line 408-409.

We found the detailed staging and timeline of polarization laid out in figure 1 to be thorough and helpful to understanding the process of gut polarization. However, figure 7 only contains images of later stages. Adding images of early stages in the double mutant (hmr-1; par-3) would be useful in connecting the phenotypes across figures.

We appreciate this feedback. We are following up with the earlier stages in more detail in our future work.

© 2022. Published by The Company of Biologists under the terms of the Creative Commons Attribution License (https://creativecommons.org/licenses/by/4.0/).

- We were curious if the gut lumen edemas, found in the various mutants, formed in stereotyped locations between pairs of intestinal cells or stochastically. For example, do they form exclusively at midlines where the lumen swells, or do some occur as ectopic lumens forming between lateral cell-cell contacts? Scoring positions of edemas could help generate hypotheses about which junctions are maintained and what exactly goes wrong when edemas form.

We examined where junctional rings were present relative to membranes in 1.5-fold embryos which showed that the rings form near the midline between cells at cell-cell contacts which is now shown in supplemental figure 9. This analysis did not reveal a stereotypical pattern of ring formation in the intestine. In future work, we hope to complete the timelapse microscopy necessary to determine how the edema position relates to the position of the pockets of local polarity in PAR-3gut(-) worms.

- There is some minor redundancy in the data shown here and in the authors' earlier work (Sallee et al. 2021):

- The aPKC gut (-) phenotypes shown in Figure 3 of this manuscript were reported in Figure 6 of Sallee et al., although they are compared to a wider variety of genetic backgrounds here. This is not really an issue, but it would be helpful if the authors would more specifically refer back to their previous work in order to put the current results in context.

We appreciate this point and have added text to more specifically compare the results reported here with those from our previous work. For example, please see lines 250-251, 276-277, and 690-691 .

- Mislocalization of LET-413 and DLG-1, reported for aPKC gut(-) and PAR-3 gut (-) in Figure 5 of this manuscript, was shown previously for PAR-6 gut(-) in Figure 5 of Sallee et al. Strictly speaking, these data are not overlapping, since different proteins were depleted, but it would be shocking if the PAR-6 and aPKC phenotypes were different since these two proteins form an obligate heterodimer. Again, it would be helpful for the reader if the results from Sallee et al. were specifically mentioned and discussed in the context of Figure 5.

We appreciate this point and have added text to more specifically compare the results reported here with those from our previous work see lines 276-277 and 690-709.

- Figure 1: A supplemental movie would help with visualizing puncta movement during polarity establishment, if feasible.

We have included three supplemental movies (1,2, and 4) to help visualize puncta movement during polarity establishment. For similar movies, please also see Achilleos et al., 2010 and Feldman and Priess, 2012.

- Figure 2 D-H'': It's unclear whether the resolution of the images allows for the distance measurements performed. From the images, the proteins look colocalized, and comparison of proteins where distance was significant (such as D) look similar to proteins where distance was statistically insignificant (such as E). Because the precise localization of these junctional proteins isn't critical to the findings of the rest of the paper, we suggest omitting the distance quantification.

We appreciate this comment, but feel that the quantification which agrees with previous reports (Segbert et al 2004) provides a more quantitative understanding of the junctional structure than has previously been published. In addition, we believe that the quantification helps to more confidently indicate the relative localization of PAR-1 and AFD-1 within apical junctions since the localization of these proteins in the intestine has not been previously examined.

© 2022. Published by The Company of Biologists under the terms of the Creative Commons Attribution License (https://creativecommons.org/licenses/by/4.0/).

- We would prefer to see the PKC-3 and PAR-3 data from figure S5 included in the main Figure 5 - these data are interesting and important because they help to clearly establish the hierarchy of polarity proteins in this tissue.

We appreciate this comment but felt it fit better in the supplemental material due to space constraints.

- Discussion: The authors write “The kinase activity of PKC-3 is thought to be inactive when in complex with PAR-3, and one role of PAR-3 may be to keep PKC-3 in an inactive state (Soriano et al., 2016).” However, the conclusions of Soriano et al. have been challenged (Holly & Prehoda, 2019). We would urge the authors to be cautious about taking sides in this debate in the absence of direct evidence from C. elegans.

We appreciate the reviewer for bringing this evidence to our attention and have edited the text appropriately.

Second decision letter

MS ID#: DEVELOP/2021/200325

MS TITLE: Separable mechanisms drive local and global polarity establishment in the C. elegans intestinal epithelium

AUTHORS: Melissa A Pickett, Maria D. Sallee, Lauren E Cote, Victor F. Naturale, Deniz Akpinaroglu, Joo Lee, Kang Shen, and Jessica Feldman
ARTICLE TYPE: Research Article

I am happy to tell you that your manuscript has been accepted for publication in Development, pending our standard ethics checks.

Reviewer 1

Advance summary and potential significance to field

Melissa Picket and collaborators have used C. elegans to examine the fine distribution of eight polarity and junctional proteins over development, as well as their requirements to establish a polarized intestinal epithelium using gut-specific depletion of several markers. Their precise mapping shows that proteins that will eventually become basolateral (LET-413, LGL-1, PAR-1, DLG-1) can be found at early stages of polarization at the midline with apical markers (PAR-3, PKC-3, HMR-1, AFD-1). They found that these markers are in order of requirements for survival and polarization PAR-3 = PKC-3 ≈ HMR-1 ≥ DLG-1 ≈ AFD-1 >> PAR-1 ≈ LGL-1. They further found that PAR-3 requirement is more important than that of PKC-3 for gut polarization, since the adherens junction proteins HMR-1 and AFD-1 could assemble in the absence of PKC-3 but not of PAR-3. Finally, they report that PAR-3 is not required for initial cellular polarization, but is essential for global polarization across the tissue, and that the combined absence of PAR-3 with E-cadherin/HMR-1 prevents local cellular polarization.

Comments for the author

I keep being very interested in the work and will again advocate that it should be published in Development, as I am overall satisfied with the authors' responses to the reviewers' comments. However, I am disappointed with their response to my first initial point about the resolution in Figure 2, which was also pointed out by the other two reviewers. I find it surprising that the authors could not find a way to improve the resolution as there are several available methods and setups besides structured illumination such as expansion microscopy, the Zeiss Airyscan technology (even deconvolution might improve), and I am also slightly surprised that the out of focus signal from the signal from the skin would so much limit the reconstruction. As the third reviewer notes, this might

© 2022. Published by The Company of Biologists under the terms of the Creative Commons Attribution License (https://creativecommons.org/licenses/by/4.0/).

not be central to the main conclusions, but I think the authors should do more than adding a limitation on lines 162-163.
I accept that they could not build a viable combination to examine LET-413 requirement, but, for the record and their future work, there are alternatives to the strategy they used.

Reviewer 2

Advance summary and potential significance to field

The authors have address all of the relatively minor concerns I had with the original submission. Congratulations on an excellent manuscript!

Comments for the author

The authors have address all of the relatively minor concerns I had with the original submission. Congratulations on an excellent manuscript!

© 2022. Published by The Company of Biologists under the terms of the Creative Commons Attribution License (https://creativecommons.org/licenses/by/4.0/).
